# Analysis Profiling of 48 Endogenous Amino Acids and Related Compounds in Human Plasma Using Hydrophilic Interaction Liquid Chromatography–Tandem Mass Spectrometry

**DOI:** 10.3390/molecules29245993

**Published:** 2024-12-19

**Authors:** Xiongwei Yin, Irene Baldoni, Erwin Adams, Ann Van Schepdael

**Affiliations:** Department of Pharmaceutical and Pharmacological Sciences, Pharmaceutical Analysis, KU Leuven, 3000 Leuven, Belgium; xiongwei.yin@kuleuven.be (X.Y.); irene.baldoni@edu.unifi.it (I.B.); erwin.adams@kuleuven.be (E.A.)

**Keywords:** amino acids, quantitative, HILIC-MS/MS, plasma, non-derivatization, surrogate matrix method

## Abstract

Analyzing and detecting endogenous amino acids in blood is of crucial importance for the diagnosis of medical conditions and scientific research. Considering the lack of UV chromophores in most of these analytes and the presence of several interfering substances in plasma, the quantification of quite a few amino acids and related compounds presents certain technical challenges. As a blank plasma matrix lacking these endogenous substances does not exist, the surrogate matrix method is used, as well as isotopic internal standards for calibration, to ensure the accuracy and reliability of the study. Method validation was conducted for 48 target analytes, giving the following results: linearity (R^2^ at least 0.99), limit of quantification (from 0.65 to 173.44 μM), precision (intra-day and inter-day RSD for LQC ranged from 3.2% to 14.2%, for MQC from 2.0% to 13.6%, and for HQC from 1.6% to 11.3%), accuracy, recovery, and stability of the method (all complied with the guidelines). To further investigate the applicability of this method to large-scale sample analysis, the method was successfully applied to the analysis of amino acids in plasma samples collected from 20 control individuals, demonstrating its wide application scope for clinical diagnosis and metabolic research.

## 1. Introduction

Taking measurements of amino acid (AA) levels in the body has many implications from a clinical diagnostic perspective, including providing useful information about metabolic status, assessing nutritional health, and evaluating the progression of diseases [1,2,3]. There is growing recognition that AAs are suitable biomarkers for the development and progression of metabolic disorders and that AA profiling during the neonatal period can help detect diseases related to amino acid metabolism, such as maple syrup urine disease [4,5] and phenylketonuria [6], at an early stage. A wide range of physiological and pathological processes are also influenced by AAs, such as in the nervous system [7], the cardiovascular system [8], immune regulation [9], and exercise recovery [10]. It has been reported that branched-chain AAs are biomarkers of depression [7], and liver damage can cause abnormal metabolism of leucine and isoleucine [11]. An indication providing insight into the condition of immune function can be found by observing changes in glutamine and arginine levels [9]. Athletes may benefit from optimizing their diet to reduce muscle damage by adjusting the intake of branched-chain AAs [12]. For this reason, it is essential to monitor AA levels in the blood regularly to assess metabolic health, control diseases, and maintain health. The complex biological matrix makes it difficult to quantify multiple AAs simultaneously, which may also influence the sensitivity and accuracy of AA analysis. Quantitatively analyzing various AAs is also challenging from the perspective of chemical analysis [13]. The first problem is that most AAs do not have a strong UV chromophore, which leads to difficulty analyzing them effectively using traditional UV or fluorescence detection methods. This makes it necessary to perform derivatization with reagents before the column, in the column, or post-column [14,15]. Because some AAs can occur as isomers, the impact on the separation and quantification of AAs is even more challenging.

As a result of the introduction of mass spectrometry (MS) as a detector, the limitations associated with amino acid detection have been greatly improved. This not only eliminates cumbersome chemical derivatization steps but also enhances the detection limit and sensitivity, making it suitable for the simultaneous quantitative analysis of many AAs simultaneously [16]. In gas chromatography–mass spectrometry (GC-MS), AAs are typically converted into volatile derivatives before being analyzed [17,18]. While derivatization is an effective method, it adds extra steps and complexity to the analysis. As an alternative, capillary electrophoresis–mass spectrometry (CE-MS) is a technique that is more selective in detecting AAs in complex matrices [19]. Analyzing plasma samples with CE-MS is difficult because of the small injection volume, the complex pretreatment steps, and the ease with which the matrix can adhere to the capillary wall, which significantly impacts sensitivity and quantitative accuracy. Liquid chromatography–mass spectrometry (LC-MS) is a highly effective method for analyzing AAs in plasma [20]. While it has excellent sensitivity and selectivity, there are still some challenges to overcome. As a result of their high polarity and water solubility, some AAs are poorly retained in conventional reverse-phase liquid chromatography (RPLC). There is a reasonable explanation for this phenomenon, namely, the weak interactions between the rather polar AAs and the hydrophobic stationary phase, which results in fast elution with the mobile phase. The variety of isoelectric points and the high acidity–alkalinity difference between AAs further complicate the separation. Recently, the use of ion-pairing agents has been introduced. However, they can cause irreversible column damage [21]. Mixed-mode chromatography (e.g., adding functional groups to the stationary phase) has been shown to enhance the interaction between AAs and non-polar stationary phases, improving AA retention [22]. The hydrophilic interaction liquid chromatography–mass spectrometry (HILIC-MS) process is also an attractive option for the analysis of compounds with polar characteristics.

This study aimed at developing a rapid HILIC-MS/MS method that would be capable of analyzing approximately 50 AAs and related compounds present in human plasma. As part of the process, the method employs activated carbon treatment to remove endogenous interfering substances from plasma samples. The results were corrected using isotope-labeled internal standards, significantly enhancing accuracy and reliability. So, the quantitative detection of AAs in human plasma samples without derivatization has been accomplished through this method, resulting in a significant reduction in sample preparation steps and an improvement in the efficiency of the AA analysis process, which shows broad application prospects in clinical diagnosis, metabolic research, and disease monitoring.

## 2. Results

### 2.1. Optimization of the Method (Selection of Stationary Phase and Mobile Phase)

The method was developed in two steps. The initial step involved selecting the stationary phase. HILIC columns are more effective than RPLC C18 columns for the separation of strongly polar compounds, such as AAs and their derivatives. In addition, HILIC mobile phases contain a high proportion of organic solvents, such as acetonitrile, which are more conducive to proper ionization in MS. Several types of HILIC columns are available, including hexagonal, bare silica, and amide-based columns. In this study, a comparison was conducted between two different HILIC columns. One was the Phenomenex Luna HILIC NH_2_ column (3.0 mm × 150 mm, 3 μm, 100 Å), which is made up of silica gel with an NH_2_ functional group. This was compared with a Waters XBridge Amide column, which utilizes ethylene bridged hybrid (BEH) technology coupled with an acetamide-group-modified stationary phase. This enables enhanced retention of polar analytes through hydrogen bonding and polar interactions, and it has a wide pH tolerance range (pH 1–11). Figure S1 depicts the results and shows that BEH–amide columns yield better peak shapes for most target compounds, with cystine and cysteine shown as examples.

Following that, a further evaluation was conducted to determine the optimal composition of the mobile phase based on the apparent pH of mobile phase A and mobile phase additives. The purpose of this study was to investigate the effects of different concentrations of ammonium formate (AMF) and formic acid (FA) under experimental conditions. AMF was used in concentrations of 0 mM, 5 mM, 10 mM, or 20 mM, while FA was investigated in concentrations of 0%, 0.1%, 0.15%, or 0.2%. Furthermore, various pH levels were examined, including 10 mM ammonium acetate (AmAc) (pH 6.9), 10 mM ammonium bicarbonate (pH 7.95), 0.01% ammonium hydroxide in combination with 10 mM AmAc (pH 8.1), and 0.001% ammonium hydroxide (pH 9.1) [23]. Through a systematic examination of these conditions, it is possible to establish a more comprehensive understanding of the influence of AMF and FA concentrations as well as other additives on the results of the experiment.

Figure S2 shows that when only FA was used at concentrations ranging from 0.1% to 0.2%, retention times of most target analytes fell between 2 and 4 min, leading to significant overlap. Under these conditions, the pH ranged between 2.4 and 2.6. Co-elution was observed for isomers, such as leucine/isoleucine and valine/norvaline. As the concentration of FA increased, the overlap in the Total Ion Chromatogram (TIC) across various ion channels became more pronounced, and retention times shifted further. When only AMF was used at concentrations of 5–20 mM with a pH of 6.5–6.8, it was found that AMF significantly suppressed the peak signals of glycine and cysteine (Figure S3). In Figure S4, the neutral and alkaline systems with 10 mM AmAc, 10 mM ammonium bicarbonate, and/or 0.001% ammonium hydroxide produced broadened, tailing peaks for some target compounds. Optimal separation of most target compounds was achieved at a pH near 3, with three specific mobile phase combinations: 5 mM AMF + 0.1% FA (pH 2.98), 10 mM AMF + 0.15% FA (pH 3.04), and 20 mM AMF + 0.2% FA (pH 3.14). These combinations provided effective separation of most target compounds. The separation and signal strength of the 3-methyl-l-histidine/1-methyl-l-histidine pair are shown in Figure S5. Under the first condition (5 mM AMF + 0.1% FA (pH 2.98)), tailing peaks were observed. Both the second (10 mM AMF + 0.15% FA (pH 3.04)) and third conditions (20 mM AMF + 0.2% FA (pH 3.14)) achieved good separation, but the second condition provided a stronger signal. Therefore, the final composition of the mobile phases was as follows: mobile phase A consisted of 95% aqueous solution of 0.15% FA, 10 mM AMF, and 5% acetonitrile; and mobile phase B consisted of 95% acetonitrile solution of 0.15% FA, 10 mM AMF, and 5% H_2_O. The detailed Multiple Reaction Monitoring (MRM) information for AAs, related compounds, and internal standards (ISs) is provided in Table 1.

The order of elution of the AAs and their derivatives was mainly determined by their polarity, the charges of functional groups (such as amino groups, carboxylic acids, and side chains), and the composition of the mobile phase. In HILIC columns, the stationary phase is commonly a polar material, while the mobile phase is high-content organic.

It should be noted that the first AAs to be eluted from the column are phenylalanine, tryptophan, leucine, isoleucine, methionine, proline, valine, and alanine. The side chains of these non-polar AAs are mainly composed of hydrophobic hydrocarbon chains, which have low polarity and interact weakly with the polar stationary phase and therefore elute first. Kynurenine is a derivative of tryptophan, and the combination of the amide group (-CONH_2_) and phenolic hydroxyl group (-OH) makes the compound more polar, so during chromatographic separation, it elutes later than tryptophan. Because the methyl group from d-alanine is replaced by a less polar cyclohexyl group in 3-cyclohexyl-d-alanine hydrate, the elution of alanine occurs before that of 3-cyclohexyl-d-alanine hydrate.

During gradient elution in HILIC, the water content in the mobile phase will increase, and the water-rich layer will be fixed on the surface of the amide groups. Those AAs with stronger hydrophilicity can form hydrogen bonds or other polar interactions with these water molecules more readily, thus allowing them to remain longer on the column. Hence, the following polar uncharged neutral AAs are subsequently eluted: tyrosine, cysteine, threonine, glycine, glutamine, serine, and asparagine. They contain side chain functional groups (such as hydroxyl groups and amide groups) that interact with water molecules. In addition to being less polar than cystine, homocystine also has a longer carbon chain, which increases the molecule’s hydrophobicity and enables it to be eluted before cystine. *N*-acetyl-l-cysteine, an acetylated molecule, is less hydrophilic and has a lower hydrogen bonding capacity, so it becomes less polar and more hydrophobic. When HILIC chromatographic separations are carried out, l-cysteine is usually eluted after the *N*-acetyl-l-cysteine (metabolite). l-(+)-α-phenylglycine contains a non-polar phenyl group (-C_6_H_5_) attached to the α-carbon of glycine, which reduces its overall polarity. As a result, it exhibits faster elution compared to glycine in HILIC chromatography. (*R*)-2-(2,5-dihydrophenyl)glycine has a partial hydrogenation of the benzene ring (2,5-dihydrophenyl). Even though it is more polar than l-(+)-phenylglycine, it is generally less polar than glycine.

Polar AAs with a negative charge like aspartic acid and glutamic acid have side chains containing carboxylate groups (-COO^−^). Because they are (to some extent) negatively charged, they exhibit strong interactions with water molecules as well as positively charged molecules. l-5-oxoproline is an intermediate product in the glutathione metabolic pathway. Because it has a relatively weak polarity due to the lactam ring in its structure, it has a shorter retention time than glutamic acid and glutamine. The following AAs are positively charged: histidine, arginine, and lysine. Their side chains contain amino or guanidino groups, which are extremely polar and result in a long retention time during chromatography. l-citrulline is a derivative of l-arginine. l-citrulline has a weaker polarity than l-arginine because of the absence of a strong polar guanidinium group, so the elution order is before l-arginine. Compared with l-lysine, adding a hydroxyl group in hydroxylysine increases the ability of the molecule to interact with water, increases its hydrophilicity and hydrogen bond formation ability, and delays the elution of hydroxylysine.

**Figure 1:**
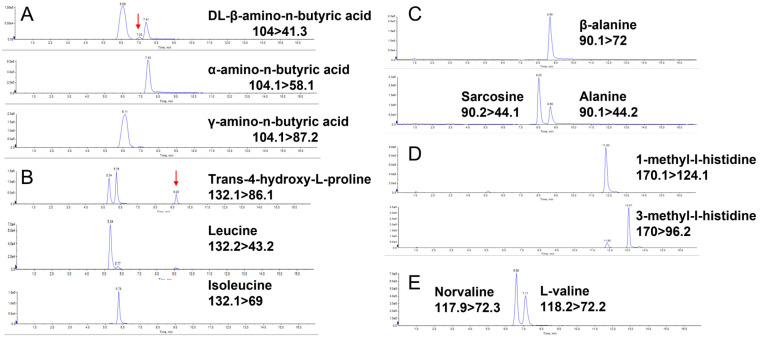
Chromatographic separation of five sets of AAs and their isomers. (**A**) dl-β-amino-n-butyric acid, α-amino-n-butyric acid, and γ-amino-n-butyric acid (*m*/*z* 104). (**B**) *Trans*-4-hydroxy-l-proline, leucine and isoleucine (*m*/*z* 132). (**C**) β-alanine, alanine and sarcosine (*m*/*z* 90). (**D**) 1-methyl-l-histidine and 3-methyl-l-histidine (*m*/*z* 170). (**E**) Norvaline and l-valine (*m*/*z* 118). The arrows denote the relevant peak.

Five sets of positional isomers exist among the 48 AAs and their derivatives. Figure 1 shows the results of their specific separation.

(1) dl-β-aminoisobutyric acid, α-amino-n-butyric acid, and γ-amino-n-butyric acid are positional isomers. It is the amino group position that distinguishes these isomers from one another. As a result, there will be a difference in their retention times during gradient elution due to this positional difference. Three MRM channels were selected to optimize the separation. dl-β-aminoisobutyric acid was channeled at 104 > 41.3; the characteristic product ion of α-aminobutyric acid was 104.1 > 58.1, and the characteristic product ion of γ-aminobutyric acid was 104.1 > 87.2. Consequently, the three positional isomers can be identified and differentiated more precisely.
(2) A similar situation is observed with leucine, isoleucine, and *trans*-4-hydroxy-l-proline. These compounds generate identical product ions following gradient elution, producing strong signals in the 132.2 > 86.1 channel. Despite this, *trans*-4-hydroxy-l-proline is completely baseline separated from leucine and isoleucine. Because leucine and isoleucine are challenging to separate under baseline conditions, a specialized ion channel is employed to distinguish them. Ultimately, channel 132.2 > 43.2 was used as the characteristic fragmentation of leucine, while channel 132.2 > 69 was recognized as the corresponding product ion for isoleucine [22]. MS distinction enabled the effective differentiation between the two AAs, thereby improving the overall separation efficiency.
(3) Because they have different amino group positions, beta-alanine and alanine are positional isomers. The beta-alanine (90.1 > 72) and alanine (90.1 > 44.2) components can be separated by selecting different product ions. Another positional isomer of alanine is sarcosine, which is formed upon methylation of the amino group of glycine. Although sarcosine possesses the same product ion as alanine, they are completely baseline separated from each other.
(4) The different methyl group position in 3-methyl-l-histidine and 1-methyl-l-histidine also makes them positional isomers. Being completely separated in a chromatogram, they can be quantified separately upon selecting different product ion channels.
(5) Aside from that, norvaline and valine are side chain isomers with different branching of the carbon chain. Although they have a common product ion (118.2 > 72.3), the two cannot be completely separated chromatographically. However, norvaline (118.2 > 55.1) exhibits a selectable characteristic product ion that allows for exact quantification.

### 2.2. Sample Preparation

An effective pre-treatment technique removes impurities from biological samples, thereby reducing matrix effects that interfere with analytical results and optimizing the extraction and recovery of target analytes. In this way, it is possible to ensure the accuracy and sensitivity of the analysis. Biological sample processing involves protein precipitation with organic solvents, liquid–liquid extraction, and/or solid-phase extraction. The removal of interfering substances from blood samples, including proteins, lipids, and steroid hormones, is necessary to optimize the ensuing analytical steps. Considering the high cost of solid-phase extraction and the complexity of liquid–liquid extraction, in this study, the plasma proteins were precipitated with organic solvents. The effects of different organic solvents (methanol, acetonitrile (ACN), isopropanol) containing 0.1% FA were assessed to optimize the precipitation conditions. The following procedure was employed for the test: 50 µL of water and 50 µL of plasma were added to 300 μL of organic solvent and shaken for 30 s. The mixture was centrifuged for 15 min at high speed (13,200× *g*) and filtered via a 0.22 μm polytetrafluoroethylene filter (PTFE) filter. The next step was a comparison of the signal response intensity of a selection of the target compounds in HILIC-MS/MS after they had been treated with different solvents (Figure S6). The results showed that 0.1% FA in acetonitrile–methanol (75:25, *v*/*v*) provided a better mass spectrometry response for most target compounds and enabled greater extraction efficiency. FA is primarily used to lower the pH of the solution to assist in the denaturation and precipitation of proteins, thus more effectively removing proteins from plasma and reducing the impact of interfering substances. Secondly, most AA samples have good solubility in ACN, and the latter is also compatible with MS. For this reason, MeOH-ACN (1:3) containing 0.1% FA was chosen as the final mixture for protein precipitation in plasma samples.

### 2.3. Stripped Plasma

As mentioned above, no blank plasma matrix free of AA analytes is available in view of their endogenous nature. Therefore, an activated carbon stripping method was utilized in this study to prepare an alternative blank matrix. A procedure from the relevant literature was used [24]. After adding 100 mg of activated carbon to 1 mL of human plasma and allowing it to stand for 8 h at 4 °C, some AAs were still detected through LC-MS analysis (see Figure S7). As a further measure to reduce the residual AA signals, the amount of activated carbon was increased to 200 mg, and the reaction time was extended to 24 h. Even though the AA signals decreased, a small number of AAs remained detectable, which is consistent with previous reports [25]. The final choice of using 100 mg of activated carbon per mL of plasma was made due to the concern that an excessive amount of activated carbon may excessively remove other components of plasma (removing lipids, cholesterol, steroid hormones, etc.), thus destroying the original plasma environment.

### 2.4. Method Validation

#### 2.4.1. Selectivity and Carry-Over

**Figure 2:**
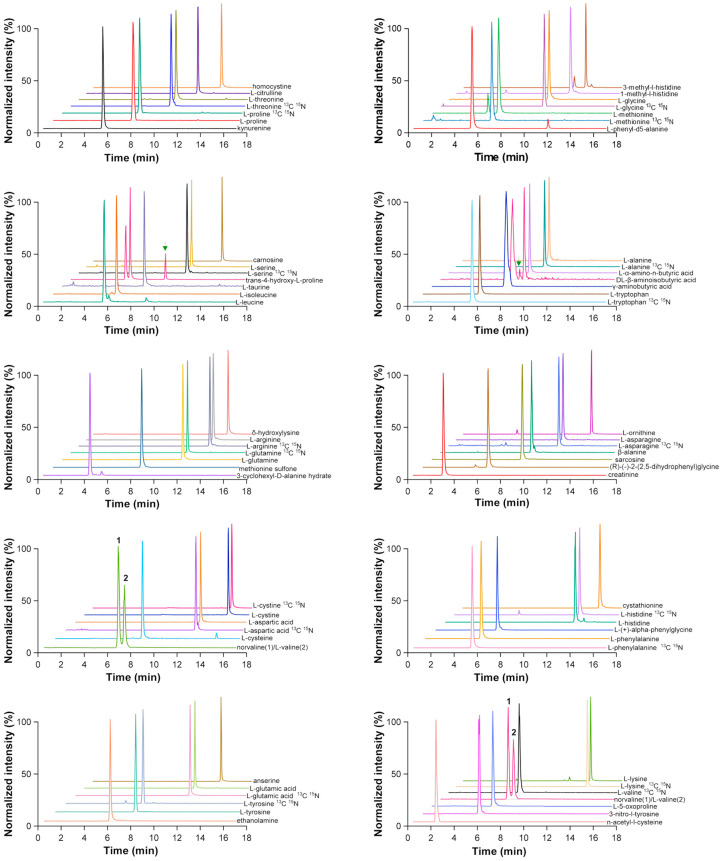
MRM for 48 AAs, related compounds, and IS in QC samples. The arrow denotes the relevant peak.

Figure 2 shows the MRM signals of 48 AAs, related compounds, and the IS in the quality control (QC) samples. The overall TIC can be seen in Figure S8. An evaluation of the carry-over of the chromatographic system has been conducted in this study. Three injections of high quality control (HQC) samples, each time followed by a blank injection, were used to determine the residual signals of 48 analytes and the IS in the blank. The results showed that the residues of the 48 analytes and the IS were all less than 20% of the lower limit of quantification (LLOQ) concentrations, while the internal standard residue was less than 5%, ensuring that the system was accurate and reliable based on the EMA guidelines for carry-over [26].

#### 2.4.2. LOD, LLOQ, and Linearity

An analysis of 48 analytes was performed using the HILIC-ESI-MS/MS system. In particular, the concentration of 17 proteinogenic AAs was determined utilizing the isotope IS method, and the concentration of the remaining 31 AAs and their derivatives was determined through l-phenyl-d5-alanine as the IS. Table 2 shows the detailed values for the limit of detection (LOD), the LLOQ, and the linearity range for all 48 analytes. From the data, it is clear that all analytes show good linear relationships after 1/X weighted linear regression within the calibrated concentration range, with determination coefficients (R^2^) of at least 0.99 for all analytes.

#### 2.4.3. Accuracy and Precision

At the three different concentration levels, namely low quality control (LQC), medium quality control (MQC), and HQC, the intra-day accuracy results for the samples were as follows (Table 3): the LQC has an accuracy range of 85.0% to 116.0%, and the relative standard deviation (RSD) is less than 14.2%; the MQC accuracy ranges between 86.8% and 112.7%, and the precision is less than 9.5%; and the HQC accuracy ranges between 86.8% and 110.1%, and the precision is less than 9.3%. In terms of the inter-day accuracy ranges of LQC, MQC, and HQC, they are 82.7–117.5%, 86.5–117.1%, and 84.8–108.6%, respectively. As for the precision, they are 3.2–13.6%, 2.0–13.6%, and 1.6–11.3%, respectively. Table 3 illustrates that the precisions at all low, medium, and high concentration levels are less than 15%, which indicates that the method is repeatable, stable, and reliable. The accuracy bias is less than 15% for each MQC and HQC concentration level and less than 20% for the inter-day LQC. So, it is a method that can be used to analyze plasma samples quantitatively.

#### 2.4.4. Matrix Effect

Results from Table 4 indicate that the plasma matrix does affect the analysis of these AAs and their related compounds. Despite this, the effects on some AAs remain within the acceptance range (80–120%) because they (l-phenylalanine, l-tryptophan, l-methionine, l-valine, l-proline, l-tyrosine, l-alanine, l-threonine, l-glycine, l-glutamine, l-serine, l-asparagine, l-glutamic acid, l-aspartic acid, l-arginine, l-lysine, l-cystine) were determined by using isotope-labeled standards as the IS. Although matrix effects interfere with the analysis, using isotope-labeled ISs effectively compensates for this effect, keeping the analytical method reliable and accurate. Some of the remaining AAs and related compounds had results greater than 120%, indicating that the plasma matrix strongly interfered with the ionization process of the analytes, resulting in an ion enhancement.

#### 2.4.5. Extraction Recovery

As shown in Table 4, the recovery test was performed for LQC, MQC, and HQC. The recoveries ranged from 81.6% to 118.1% for the LQC, 81.3% to 118.8% for the MQC, and 82.4–114.4% for the HQC. It was found that the RSDs for most samples were less than 15% at the three concentrations. As can be seen from these results, the recoveries were generally stable and within an acceptable range at different concentrations.

#### 2.4.6. Stability

In this experiment, the samples were evaluated for their 4 °C stability and three cycles of freezing. According to the results of the stability test conducted at 4 °C (Table 5), the following was observed: the stability results for the LQC, MQC, and HQC samples were 88.5–116.5%, 85.2–118.7%, and 84.3–110.0%, respectively, with RSDs of 3.4–13.9%, 2.4–12.6%, and 1.5–7.5%, respectively. As a result of three cycles of freezing and thawing, the following stabilities were obtained for the LQC, MQC, and HQC samples, respectively: 83.5–114.9%, 86.1–115.1%, and 84.0–113.5% and RSD 3.9–15.4%, 1.8–11.1%, and 0.9–11.9%, respectively. Table 5 demonstrates that plasma samples at three different concentrations were stable. The stability of all biological samples’ extracts was not examined because they were measured immediately following preparation.

#### 2.4.7. Dilution Integrity

Six samples were processed in parallel to assess any dilution effect using a high-concentration quality control sample (HQC) with a 20 times higher concentration added to the blank matrix. Table 5 shows the results obtained after a 20-fold dilution of this sample, exceeding the upper limit of quantification (ULOQ). As shown, all target analytes remained accurate and precise within 15%, indicating that the method did not produce significant bias during the dilution process and verifying its reliability for processing samples outside of the linear range of the method.

### 2.5. Comparison with Other Analytical Methods

The determination of AAs in biological fluids has been extensively studied, and reverse-phase high-performance liquid chromatography–mass spectrometry is a popular analytical technique. As AAs have poor retention on C18 columns, this must generally be improved through derivatization or ion pairing. Although such a technique has been used for the quantification of 30 AAs in plasma, the irreversible change induced by ion-pairing reagents in the column limits its practical application [27]. Based on the existing literature, HILIC-MS/MS bioanalytical methods are mostly used to quantify multiple AAs simultaneously in urine [28], sweat [29], and cell extracts [30]. The standard addition method has the advantage that it is considered a very accurate quantitative method, but it is labor-intensive, making it difficult to apply to analyses of large sample sets [31]. It is important to understand how to solve the problem of a blank matrix when analyzing AAs in plasma because AAs are endogenous compounds. For example, Hu et al. used 4% bovine serum albumin as a substitute matrix [32], and Chen et al. used acetonitrile as a surrogate matrix [33]. None of these studies used isotope-labeled internal standards to determine AAs in plasma. There have also been targeted studies on the detection of specific AAs. For example, a plasma matrix lacking thiol AAs can be prepared using a reagent containing a maleimide group, which reacts with thiols at pH 7. In that way, thiol-containing AAs could be determined in plasma [20]. Nevertheless, these methods usually target only one type of amino acid, making it difficult to obtain comprehensive coverage of AAs. Our research covers an extensive range of AAs and their derivatives (nearly 50 AAs and related compounds). Activated carbon may be more advantageous than previous methods in stripping plasma to obtain a blank matrix and using isotope-labeled standards as internal standards for some AAs. Analyzing biological samples with a simplified pre-processing method will provide a more accurate and comprehensive solution to detect AAs in biological samples.

### 2.6. HILIC-MS/MS Analysis of Samples

**Figure 3:**
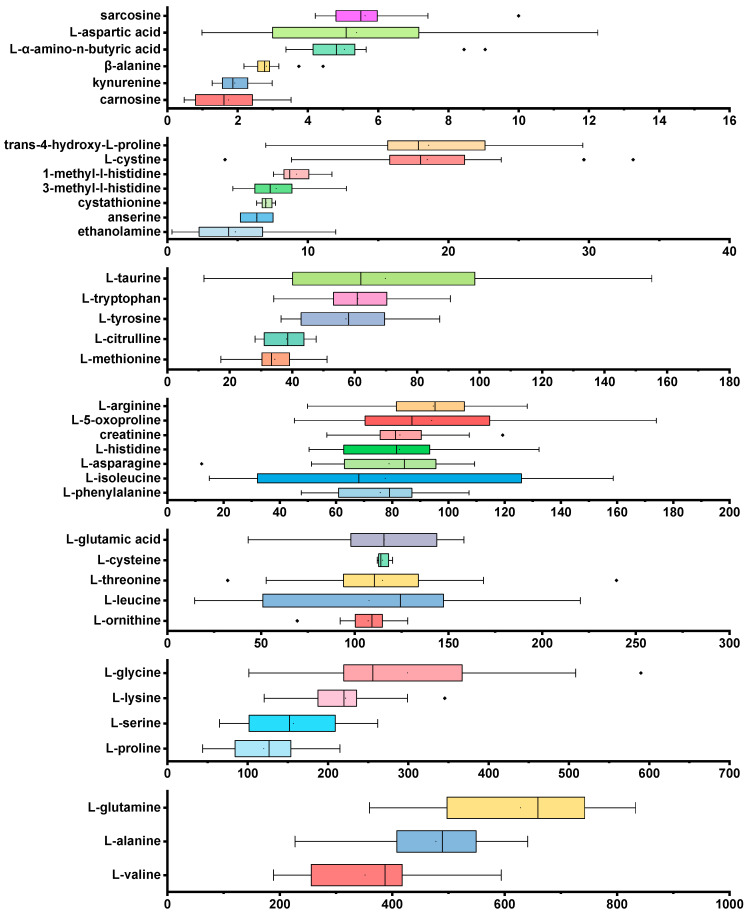
Concentrations of AAs and related compounds detected in 20 controls (μM).

As part of this study, the above AA analysis method was applied to accurately determine the concentration of various AAs and their related compounds in human plasma samples. It has been demonstrated that the method is highly sensitive and specific and capable of detecting a variety of endogenous AAs in a complex plasma matrix. Figure 3 provides a detailed illustration of the concentration distribution of AAs and related compounds in the plasma of 20 subjects. It was found that the concentrations of different AAs and metabolites in plasma vary widely, ranging from a few micromolars (μM) to hundreds of micromolars. This is indicative of a wide range of AA concentrations in blood, which is affected by differences in metabolic needs, nutritional status, and physiological functions among individuals. Box plots provide a more intuitive representation of concentration distributions for these AAs and related compounds. As a result of the analysis, compared with other studies [34,35,36,37], the 37 detected AAs generally fall within the reference range. l-ornithine, l-leucine, l-threonine, l-cysteine, l-glutamic acid, l-proline, l-serine, l-lysine, l-glycine, l-valine, l-alanine, and l-glutamine were found to be relatively high in the samples. In addition to their essential roles in protein synthesis and metabolic regulation, these AAs also reflect human nutritional status, energy metabolism, and overall physical well-being. It has been demonstrated that this method, following its development and validation, is reliable and sensitive when tested on plasma samples. Using the above AA analysis method to detect these concentrations not only serves as an important means to assess metabolic status in individuals but could also be used to detect biomarkers associated with metabolic disorders. In addition, it aids in the early detection, diagnosis, and treatment of metabolic disorders and provides scientific support for personalized medicine and precision nutritional interventions.

## 3. Materials and Methods

### 3.1. Reagents and Chemicals

Activated charcoal powder was purchased from Sigma-Aldrich (Saint Louis, MO, USA). Hydrochloric acid (HCl, 37%) was sourced from Fisher Scientific (Merelbeke, Belgium). Methanol (MeOH, LC-MS grade) and H_2_O (LC-MS grade) were bought from Fisher Scientific (Loughborough, UK). Acetonitrile and isopropanol were purchased from Biosolve Chimie SARL (Dieuze, France.) The following compounds were purchased from Sigma-Aldrich: l-anserine, l-alanine, l-arginine, l-aspartic acid, l-asparagine, l-carnosine, l-creatinine, l-citrulline, l-cystine, cystathionine, ethanolamine, l-glutamine, l-glutamic acid, l-glycine, l-histidine, l-isoleucine, l-leucine, l-lysine, l-methionine, l-methionine sulfone, l-phenylalanine, l-proline, l-serine, l-threonine, l-tyrosine, taurine, l-tryptophan, l-valine, β-alanine, l-α-amino-n-butyric acid, γ-amino-n-butyric acid, dl-β-aminoisobutyric acid, l-homocystine, δ-hydroxylysine, *trans*-4-hydroxy-l-proline, 1-methyl-l-histidine, 3-methyl-l-histidine, l-ornithine, sarcosine, 3-nitro-l-tyrosine, l-5-oxoproline, and kynurenine. 3-cyclohexyl-d-alanine hydrate was obtained from Fluka Analytical (Bucharest, Romania), and l-cysteine was obtained from Federa SC SV (Brussels, Belgium). *N*-acetyl-l-cysteine was sourced from Acros Organics (Geel, Belgium). (*R*)-(−)-2-(2,5-dihydrophenyl) glycine and l-(+)-alpha-phenylglycine were purchased from Janssen (Beerse, Belgium). l-norvaline was obtained from the Tokyo chemical industry (Tokyo, Japan). AMF was bought from Sigma-Aldrich. The LC-MS FA was obtained from Merck Millipore (Darmstadt, Germany). Two internal standards were used. One is a Cell-Free Amino Acid Mixture—^13^C,^15^N (mixture of isotope-labeled ISs), which was purchased from Sigma, and the other is l-phenyl-d5-alanine (IS), which was obtained from Sigma-Aldrich. Furthermore, 0.22 μm PTFE filters were bought from BGB Analytics (Harderwijk, The Netherlands).

### 3.2. Human Plasma

The control plasma samples (EDTA plasma) originated from University Hospitals Leuven (study approval number S50495).

### 3.3. Stock Solution Preparation

Asparagine, *N*-acetyl-l-cysteine, l-(+)-α-phenyl glycine, (*R*)-(−)2-(2,5-dihydrophenyl) glycine, norvaline, methionine sulfone, 3-nitro-l-tyrosine, and l-glutamine were dissolved in 0.1 M HCl to formulate a solution with a concentration of 10 mM. Glycine and l-taurine were prepared by dissolving them in 1 M HCl. Kynurenine and 3-cyclohexyl-d-alanine were dissolved in 0.1 M HCl to obtain a solution with a concentration of 5 mM. l-phenylalanine, l-leucine, l-tryptophan, l-isoleucine, l-5-oxoproline, l-valine, l-proline, γ-aminobutyric acid, l-tyrosine, l-taurine, β-alanine, l-alanine, *trans*-4-hydroxy-l-proline, l-threonine, l-serine, l-glutamic acid, l-citrulline, l-aspartic acid, l-histidine, l-arginine, l-lysine, l-ornithine, l-cystine, l-methionine, carnosine, 3-methyl-l-histidine, dl-β-aminoisobutyric acid, l-α-amino-n-butyric acid, creatinine, homocysteine, δ-hydroxylysine, cystathionine, 1-methyl-l-histidine, sarcosine, anserine, and ethanolamine were prepared in 0.1 M HCl to obtain a solution with a concentration of 20 mM. The low, medium, and high quality control samples (LQC, MQC, HQC) are described under the section Supporting Information Table S1.

### 3.4. Sample Preparation

The processing of real plasma samples was carried out as follows. To precipitate proteins, 50 µL of plasma and 50 µL of H_2_O were mixed with 292 µL of MeOH-ACN (1:3) containing 0.1% FA, along with 4 µL of isotope-labeled IS mixture and 4 µL of l-phenyl-d5-alanine solution. The mixture was shaken for 30 s, centrifuged for 15 min at 13,200× *g*, and filtered via a 0.22 μm PTFE filter.

### 3.5. Stripped Plasma

The blank plasma solution was obtained after two centrifugations. The plasma was first centrifuged at low speed with 4500 rpm (3645× *g*) for 30 min using a Jouan series centrifuge (Thermo Scientific, Merelbeke, Belgium), and the supernatant was reserved for later use. Then, blank-stripped plasma was prepared by mixing 100 mg of activated charcoal with 1 mL of the supernatant plasma, following the laboratory’s previous protocol [38]. The mixture was shaken at 4 °C for 8 h at 1400 rpm using a Thermomix Comfort (Eppendorf, Aarschot, Belgium), followed by centrifugation at 14,100× *g* for 30 min using a Minispin Plus centrifuge (Eppendorf). The supernatant was then stored at −20 °C for future use.

### 3.6. HILIC-ESI-MS/MS Analysis

All of the samples were analyzed with an Agilent 1100 high-performance liquid chromatography (HPLC) system (Agilent Technologies, Santa Clara, CA, USA) using an XBridge BEH Amide column (2.1 mm × 150 mm, 3.5 μm, 130 Å) paired with a protective XBridge BEH Amide Van Guard Cartridge (2.1 mm × 5 mm) (Waters, Milford, MA, USA).

The column temperature was precisely maintained at 35 °C, while the autosampler was kept at 4 °C. The injection volume for each run was set at 5 μL. The mobile phase preparation was based on a previously published article, with slight modifications [39]. Mobile phase A consisted of 10 mM AMF containing 0.15% *v*/*v* FA in LC-MS-grade water and acetonitrile (95:5, *v*/*v*), with an apparent pH of 3, while mobile phase B was made up of 10 mM AMF containing 0.15% *v*/*v* FA and acetonitrile (5:95, *v*/*v*). The elution gradient was applied as follows: 0–8 min, 95–82.5% B, 8–12 min, 82.5–70.5% B, 12-14 min, 70.5–55% B, 14–15.5 min, the system remained at 55% B, 15.5–15.6 min, 55–95% B, and 15.6–17.0 min, the system was maintained at 95% mobile phase B. The HILIC-ESI-MS/MS system was equipped with an electrospray ionization (ESI) source coupled with an AB Sciex API 3000 triple quadrupole mass spectrometer (AB Sciex Instruments, Framingham, MA, USA). The MS setup parameters were configured as follows: the ion source temperature was maintained at 450 °C, and the ion spray voltage was adjusted to 4.5 kV. The nitrogen nebulizing, curtain, and collision gas flow rates were set to 10.00, 8.00, and 10.00 arbitrary units, respectively. At the same time, the MS was configured to run in positive ion mode, utilizing MRM mode, and all of the sample data acquisition parameters for the chromatography and instrument control were handled using Analyst 1.4.3 software (AB Sciex).

### 3.7. Method Validation

The method validation was inspired by the EMA guidance and recommendations for bioanalytical method validation, covering parameters like specificity, carry-over, linearity, LOD, LLOQ, precision, accuracy, recovery, matrix effect, stability, and dilution integrity [26].

#### 3.7.1. Selectivity and Carry-Over

Because AAs are commonly present in real plasma samples and challenging to remove entirely, obtaining a true matrix free of analytes remains difficult. During method validation, the purpose of the evaluation of selectivity is to verify the ability to perform analytes’ and isotope-labeled internal standards’ identification and quantitation in the presence of other constituents (impurities, degradation products, or matrix components) and avoid interferences in retention times between analytes and isotope-labeled standards.

As for the carry-over, when triplicate injections of HQC samples are performed, followed by a blank sample, carry-over in the blank sample should not exceed 20% of the LLOQ and 5% for the IS.

#### 3.7.2. LOD, LLOQ, and Linearity

In bioanalytical methods, the LOD is defined as the point at which the signal-to-noise ratio (S/N) is greater than or equal to 3. The LLOQ is defined as S/N ≥ 10. All analytes were mixed and diluted stepwise into 7 or 8 different concentrations. A linear fit was made to the concentration using the ratio of the analyte’s peak area to the IS’s peak area conforming to 1/x. The AAs were divided into 5 groups to perform the linear regression (for detailed information, see the supporting information).

#### 3.7.3. Accuracy and Precision

For intra-day accuracy and precision of testing, three concentrations (LQC, MQC, and HQC) were injected 6 times in one day to evaluate the accuracy and precision. Inter-day accuracy and precision were determined by repeating the intra-day validation procedure on three consecutive days. Accuracy as a percentage of the nominal concentration needs to be within ±20%. The precision is expressed as the relative standard deviation (%RSD) and should not exceed 20%.

#### 3.7.4. Matrix Effect

QC samples were prepared at three different concentrations, low, medium, and high, with six parallel samples at each concentration. Matrix effects were calculated from the peak area ratio obtained when spiking the analytes into the plasma matrix after extraction divided by the peak area ratio obtained for each analyte dissolved in the neat solvent. The ratio must be within the range of 80 to 120%, demonstrating that matrix effects do not significantly impact the accuracy of the method.

#### 3.7.5. Extraction Recovery

The recovery rate calculation is based on the ratio before and after extraction, which means the peak area ratio obtained for the analytes spiked into the plasma matrix and then extracted divided by the peak area ratio obtained for the analytes spiked into the plasma matrix after extraction. Three sample concentrations were prepared, low, medium, and high, with six parallel samples for each concentration group.

#### 3.7.6. Stability

The experiment examined six parallel samples of each of three concentration levels (low, medium, and high) under two different storage conditions for stability. One was storage at 4 °C for 24 h, and the other was three cycles of freezing and thawing.

## 4. Conclusions

In this study, a reliable and efficient HILIC-MS/MS analytical method has been developed. This method can quantitatively detect 48 endogenous AAs and related compounds in human plasma within 17 min. It requires no complex derivatization reaction and only simple sample pretreatment. It has been successfully applied to the analysis of plasma samples from 20 individuals, resulting in a significant improvement in the operational efficiency of plasma sample analysis in clinical settings. The use of isotope ISs for calibration ensures the test results are highly accurate. A series of experimental results obtained during method verification indicates that the method can stably and reliably detect multiple AAs in plasma, making it suitable for metabolic AA analysis in clinical diagnosis and biomedical research, with broad applications.

## Figures and Tables

**Table 1:** Mass spectrometry parameters of AAs, their related compounds, and corresponding internal standards.

Amino Acids and Related Compounds	DP ^1^ (Volts)	FP ^2^ (Volts)	EP ^3^ (Volts)	Precursor Ion	Product Ion	CE ^4^ (Volts)	CXP ^5^ (Volts)	RT ^6^ (min)
				(*m*/*z*)	(*m*/*z*)			
*N*-acetyl-l-cysteine	19	195	5	164	122 ^a^	11	6	1.56
					76 ^b^	26	6	
creatinine	47	259	11	114	44.1 ^a^	31	7	2.41
					86.1 ^b^	17	7	
3-cyclohexyl-d-alanine hydrate	34	200	9	172.3	126.3 ^a^	18	8	4.62
					109.1 ^b^	25	8	
					83.1 ^b^	29	12	
3-nitro-l-tyrosine	30	190	9	227	181.1 ^a^	19	10	4.62
					168.1 ^b^	25	8	
l-phenylalanine ^13^C ^15^N	32	225	6	176.2	129.2 ^a^	17	6	4.65
l-phenylalanine	32	225	6	166.1	120.2 ^a^	17	6	4.67
					103.1 ^b^	35	8	
l-tryptophan ^13^C ^15^N	52	215	11	217.9	200 ^a^	14	12	4.69
l-phenyl-d5-alanine	65	140.3	7	171.3	125.2 ^a^	19	7	4.69
					154.3 ^b^	15	9	
l-tryptophan	52	215	11	205.2	188 ^a^	14	12	4.72
					146.2 ^b^	23	8	
l-5-oxoproline	48	240	9	130.1	84.1 ^a^	18	14	4.72
					56.1 ^b^	37	8	
kynurenine	32	234	11	209.1	192.1 ^a^	12	12	4.79
					94.1 ^b^	22	4	
l-leucine	20	193	9	132.2	43.2 ^a^	37	7	4.86
					86.1 ^b^	16	7	
l-isoleucine	29	234	7	132.1	69 ^a^	23	6	5.27
					86.2 ^b^	16	15	
l-(+)-alpha-phenylglycine	17	199	8	152.2	135 ^a^	14	7	5.38
ethanolamine	20	150	7	62.1	44.1 ^a^	14	7	5.41
					45 ^b^	15	6	
					62 ^b^	5	11	
(*R*)-(−)-2-(2,5-dihydrophenyl) glycine	31	265	10	154.2	91.1 ^a^	22	4	5.48
					137.1 ^b^	12	8	
l-methionine ^13^C ^15^N	55	192	11	155.8	109.1 ^a^	14	9	5.76
l-methionine	55	192	11	150.2	104.1 ^a^	14	9	5.77
					133 ^b^	13	7	
norvaline	21	220	10	117.9	72.3 ^a^	13	13	6.07
					55.1 ^b^	32	4	
γ-aminobutyric acid	33	129	7	104.1	87.2 ^a^	15	7	6.13
					69.1 ^b^	21	12	
l-valine	27	153	7	118.2	72.2 ^a^	15	13	6.55
					55.1 ^b^	29	9	
l-valine ^13^C ^15^N	27	153	7	123.9	77.1 ^a^	15	13	
l-proline	37	227	7	116.2	70.2 ^a^	21	12	6.79
					43.2 ^b^	34	10	
l-proline ^13^C ^15^N	37	227	7	122	75.1 ^a^	21	12	6.78
dl-β-aminoisobutyric acid	30	250	10	104	41.3 ^a^	37	6	6.81
					58.1 ^b^	14	4	
l-tyrosine	32	194	9	182.1	136.2 ^a^	17	8	6.82
					123.2 ^b^	27	10	
l-tyrosine ^13^C ^15^N	32	194	9	191.9	145 ^a^	17	8	6.84
l-taurine	52	211	11	126.1	107.9 ^a^	15	6	7.11
					126.1 ^b^	8	5	
l-cysteine	28	175	9	122.1	76.1 ^a^	18	14	7.50
					59.1 ^b^	32	5	
methionine sulfone	20	200	8	182.1	56 ^a^	15	16	7.59
					136 ^b^	5	6	
l-α-amino-n-butyric acid	29	197	6	104.1	58.1 ^a^	14	9	7.66
					41.2 ^b^	40	6	
sarcosine	28	212	7	90.2	44.1 ^a^	16	7	8.02
					72 ^b^	6	12	
β-alanine	25	159	7	90.1	72 ^a^	11	12	8.35
					30.1 ^b^	27	4	
l-alanine ^13^C ^15^N	40	175	10	94.1	47.1 ^a^	16	7	8.65
l-alanine	40	175	10	90.1	44.2 ^a^	16	7	8.67
					62.1 ^b^	9	12	
*trans*-4-hydroxy-l-proline	26	158	10	132.1	86.1 ^a^	18	7	8.76
					68.2 ^b^	28	6	
l-threonine ^13^C ^15^N	38	141	6	124.8	78 ^a^	16	12	9.28
l-threonine	38	141	6	120.2	74.2 ^a^	16	12	9.30
					102 ^b^	12	16	
l-glycine ^13^C ^15^N	31	172	5	79.1	32.1 ^a^	17	11	9.58
l-glycine	31	172	5	76	30.1 ^a^	17	11	9.60
					48.1 ^b^	7	7	
l-glutamine	20	245	9	147.1	84 ^a^	25	4	10.39
					130.2 ^b^	14	8	
l-glutamine ^13^C ^15^N	20	245	9	153.9	89 ^a^	25	4	10.42
l-serine ^13^C ^15^N	30	123	10	110.1	92.2 ^a^	13	4	10.43
l-serine	30	123	10	106.1	88.1 ^a^	13	4	10.45
					60.1 ^b^	15	11	
l-asparagine ^13^C ^15^N	27	205	9	139	76.9 ^a^	22	13	10.64
l-asparagine	27	205	9	133.1	74 ^a^	22	13	10.67
					87.1 ^b^	14	4	
l-glutamic acid ^13^C ^15^N	28	193	9	154.3	136.3 ^a^	13	7	10.90
l-glutamic acid	28	193	9	148.2	130.1 ^a^	13	7	10.93
					84.1 ^b^	24	15	
l-citrulline	31	177	8	176.1	159.1 ^a^	14	9	11.24
					70 ^b^	36	13	
1-methyl-l-histidine	29	220	11	170.1	124.1 ^a^	27	7	11.59
					83 ^b^	28	14	
					96.1 ^b^	30	5	
l-aspartic acid ^13^C ^15^N	29	157	8	139.1	77 ^a^	8	14	11.99
l-aspartic acid	29	157	8	134.1	74.1 ^a^	14	17	12.02
					88.1 ^b^	8	14	
l-histidine	30	150	8	156.1	110.1 ^a^	20	6	12.61
					83.1 ^b^	38	6	
l-histidine ^13^C ^15^N	30	150	8	165.3	118.1 ^a^	20	6	12.61
3-methyl-l-histidine	45	200	10	170	96.2 ^a^	23	5	12.83
					126.2 ^b^	19	7	
					109.1 ^b^	23	6	
l-arginine ^13^C ^15^N	36	155	11	184.7	75.1 ^a^	29	12	12.91
l-arginine	36	155	11	175.2	70.2 ^a^	29	12	12.92
					116.1 ^b^	20	6	
l-lysine ^13^C ^15^N	25	177	9	155.2	90.1 ^a^	23	8	13.31
l-lysine	25	177	9	147.3	84.2 ^a^	23	8	13.33
					130.1 ^b^	13	7	
homocystine	23	200	11	269.1	136 ^a^	14	8	13.37
					88.2 ^b^	34	8	
l-ornithine	31	214	7	133.2	70.2 ^a^	24	12	13.41
					116.1 ^b^	13	6	
anserine	23	209	9	241.3	109.1 ^a^	36	6	13.43
carnosine	29	210	8	227.1	110.2 ^a^	34	5	13.47
					210 ^b^	16	13	
δ-hydroxylysine	20	180	11	163	128.2 ^a^	17	9	14.09
					82 ^b^	23	12	
cystathionine	35	185	11	223	88.1 ^a^	51	8	14.33
					134 ^b^	24	10	
l-cystine ^13^C ^15^N	31	238	9	249	156.1 ^a^	18	9	14.49
l-cystine	31	238	9	241.1	152.1 ^a^	18	9	14.50
					74.1 ^b^	43	13	

^1^ DP declustering potential. ^2^ FP focusing potential. ^3^ EP entrance potential. ^4^ CE collision energy. ^5^ CXP collision cell exit potential. ^6^ RT retention time. ^a^ Represents the selected ion for quantification. ^b^ Represents the selected ion for confirmation.

**Table 2:** Linear range, LLOQ, and LOD of AAs and their related compounds.

Amino Acids and Related Compounds	LOD (μM)	LLOQ (μM)	Linearity Range (μM)	Determination Coefficient
*N*-acetyl-l-cysteine	0.87	1.73	1.73–222	0.990
creatinine	0.65	1.95	1.95–250	0.996
3-cyclohexyl-d-alanine hydrate	0.17	0.87	0.87–111	0.996
3-nitro-l-tyrosine	1.73	3.47	3.47–222	0.992
l-phenylalanine	0.33	0.65	0.65–250	0.995
l-tryptophan	1.95	3.90	3.9–250	0.997
l-5-oxoproline	2.89	5.78	5.78–2220	0.997
kynurenine	0.87	1.73	1.73–111	0.994
l-leucine	0.65	1.95	1.95–250	0.992
l-isoleucine	1.95	3.90	3.9–250	0.992
l-(+)-alpha-phenylglycine	0.58	1.73	1.73–222	0.996
ethanolamine	0.98	1.95	1.95–250	0.998
(*R*)-(−)-2-(2,5-dihydrophenyl)glycine	0.87	1.73	1.73–222	0.997
l-methionine	1.95	3.90	3.90–250	0.991
norvaline	0.87	1.73	1.73–222	0.994
γ-aminobutyric acid	1.95	3.90	3.9–250	0.996
l-valine	0.49	1.95	1.95–250	0.994
l-proline	0.49	1.95	1.95–250	0.999
dl-β-aminoisobutyric acid	3.91	7.81	7.81–250	0.994
l-tyrosine	1.95	3.91	3.91–250	0.999
l-taurine	1.73	3.47	3.47–222	0.997
l-cysteine	34.69	69.38	69.38–2220	0.995
methionine sulfone	3.47	6.94	6.94–222	0.996
l-α-amino-n-butyric acid	0.65	1.95	1.95–250	0.994
sarcosine	0.65	1.95	1.95–250	0.995
β-alanine	1.95	3.90	3.90–250	0.998
l-alanine	1.95	3.90	3.9–250	0.996
*trans*-4-hydroxy-l-proline	0.65	1.95	1.95–250	0.998
l-threonine	0.65	1.95	1.95–250	0.993
l-glycine	86.70	173.44	173.44–11,100	0.995
l-glutamine	0.58	1.73	1.73–222	0.992
l-serine	0.65	1.95	1.95–250	0.994
l-asparagine	0.58	1.73	1.73–222	0.990
l-glutamic acid	0.65	1.95	1.95–250	0.991
l-citrulline	0.98	1.95	1.95–250	0.999
1-methyl-l-histidine	0.98	1.95	1.95–250	0.993
l-aspartic acid	1.95	3.90	3.90–250	0.995
l-histidine	1.95	3.90	3.90–250	0.993
3-methyl-l-histidine	0.65	1.95	1.95–250	0.998
l-arginine	0.65	1.95	1.95–250	0.999
l-lysine	0.65	1.95	1.95–250	0.990
homocystine	1.95	3.90	3.90–250	0.997
l-ornithine	0.98	1.95	1.95–250	0.990
anserine	0.98	1.95	1.95–250	0.993
carnosine	0.65	1.95	1.95–250	0.997
δ-hydroxylysine	3.91	7.81	7.81–250	0.996
cystathionine	1.95	3.90	3.90–250	0.993
l-cystine	0.65	1.95	1.95–250	0.993

**Table 3:** Intra-day and inter-day precision and accuracy of the analyzed AAs and their related compounds.

Amino Acids and Related Compounds			Intra-Day		Inter-Day	
		Concentration (μM)	Accuracy	RSD	Accuracy	RSD
			(%)	(%)	(%)	(%)
*N*-acetyl-l-cysteine	LQC	1.73	103.0%	14.2%	90.4%	8.6%
	MQC	111.00	111.2%	7.8%	92.5%	8.6%
	HQC	222.00	86.8%	4.1%	102.4%	3.9%
creatinine	LQC	1.95	94.9%	8.3%	96.1%	10.9%
	MQC	125.00	92.4%	7.2%	91.9%	5.9%
	HQC	250.00	89.4%	5.5%	102.7%	3.2%
3-cyclohexyl-d-alanine hydrate	LQC	0.87	114.0%	6.3%	106.5%	6.8%
	MQC	55.50	91.0%	7.8%	100.9%	4.5%
	HQC	111.00	98.6%	9.3%	88.2%	4.1%
3-nitro-l-tyrosine	LQC	3.47	109.8%	8.5%	106.9%	9.9%
	MQC	111.00	94.4%	3.4%	104.1%	5.5%
	HQC	222.00	97.0%	4.4%	99.3%	5.4%
l-phenylalanine	LQC	1.95	102.3%	9.0%	97.8%	10.9%
	MQC	125.00	101.1%	4.4%	102.3%	12.4%
	HQC	250.00	105.4%	6.9%	107.1%	7.3%
l-tryptophan	LQC	3.91	112.9%	6.4%	110.7%	11.0%
	MQC	125.00	105.1%	4.2%	105.9%	3.6%
	HQC	250.00	107.4%	7.0%	102.2%	8.9%
l-5-oxoproline	LQC	5.78	114.4%	13.9%	106.9%	11.9%
	MQC	1110.00	106.5%	6.7%	102.5%	7.9%
	HQC	2220.00	109.9%	3.1%	97.3%	7.3%
kynurenine	LQC	1.73	99.9%	7.3%	116.1%	3.2%
	MQC	55.50	103.4%	6.7%	101.7%	4.3%
	HQC	111.00	106.8%	4.0%	108.6%	3.7%
l-leucine	LQC	1.95	97.2%	8.9%	90.3%	8.3%
	MQC	125.00	110.6%	7.6%	97.1%	5.8%
	HQC	250.00	93.5%	4.1%	92.4%	7.1%
l-isoleucine	LQC	3.91	86.2%	4.3%	99.0%	5.7%
	MQC	125.00	96.9%	7.8%	101.4%	9.0%
	HQC	250.00	95.2%	4.4%	104.3%	5.6%
l-(+)-alpha-phenylglycine	LQC	1.73	106.1%	6.6%	109.0%	6.4%
	MQC	111.00	90.9%	7.3%	100.2%	6.5%
	HQC	222.00	96.3%	3.7%	96.5%	3.7%
ethanolamine	LQC	1.95	110.5%	8.6%	90.7%	9.1%
	MQC	125.00	102.9%	5.6%	92.4%	7.4%
	HQC	250.00	97.3%	4.0%	95.7%	9.1%
(*R*)-(−)-2-(2,5-dihydrophenyl)glycine	LQC	1.73	99.0%	6.1%	99.8%	6.0%
	MQC	111.00	94.5%	4.0%	100.7%	2.0%
	HQC	222.00	92.9%	2.7%	106.8%	5.1%
l-methionine	LQC	3.91	107.1%	5.0%	106.7%	8.9%
	MQC	125.00	112.6%	6.4%	102.9%	7.0%
	HQC	250.00	99.2%	4.8%	94.4%	6.0%
norvaline	LQC	1.73	93.3%	10.7%	95.9%	8.3%
	MQC	111.00	98.9%	8.2%	97.0%	3.8%
	HQC	222.00	96.1%	3.4%	106.0%	3.0%
γ-aminobutyric acid	LQC	3.91	87.9%	7.8%	101.0%	8.4%
	MQC	125.00	101.9%	4.7%	86.5%	6.0%
	HQC	250.00	95.6%	8.8%	86.8%	10.8%
l-valine	LQC	1.95	85.8%	6.2%	82.7%	7.7%
	MQC	125.00	102.5%	8.5%	96.7%	8.7%
	HQC	250.00	94.3%	7.4%	91.8%	11.3%
l-proline	LQC	1.95	92.7%	8.0%	94.5%	9.6%
	MQC	125.00	86.8%	3.5%	99.4%	7.0%
	HQC	250.00	99.0%	7.0%	105.8%	8.7%
dl-β-aminoisobutyric acid	LQC	7.81	92.9%	10.4%	92.3%	7.7%
	MQC	125.00	96.3%	4.4%	88.4%	5.8%
	HQC	250.00	97.3%	3.5%	91.2%	5.2%
l-tyrosine	LQC	3.91	115.6%	6.0%	91.8%	13.4%
	MQC	125.00	105.5%	2.1%	100.9%	5.7%
	HQC	250.00	108.6%	7.8%	94.7%	4.3%
l-taurine	LQC	3.47	90.5%	5.8%	87.2%	6.3%
	MQC	111.00	98.7%	8.9%	97.3%	3.6%
	HQC	222.00	97.2%	4.2%	95.6%	4.5%
l-cysteine	LQC	69.38	88.1%	4.3%	87.8%	9.3%
	MQC	1110.00	104.6%	8.3%	90.9%	10.0%
	HQC	2220.00	107.6%	6.3%	94.1%	8.2%
methionine sulfone	LQC	6.94	100.5%	9.2%	100.9%	12.0%
	MQC	111.00	109.9%	7.9%	95.3%	8.5%
	HQC	222.00	96.0%	5.5%	105.2%	5.8%
l-α-amino-n-butyric acid	LQC	1.95	89.0%	11.9%	102.5%	9.7%
	MQC	125.00	102.3%	7.9%	96.7%	6.0%
	HQC	250.00	97.2%	4.3%	99.7%	4.7%
sarcosine	LQC	1.95	115.9%	10.2%	108.7%	6.0%
	MQC	125.00	98.8%	6.9%	94.6%	8.8%
	HQC	250.00	97.2%	3.6%	98.7%	4.7%
β-alanine	LQC	3.91	88.8%	6.8%	87.1%	5.4%
	MQC	125.00	112.0%	7.6%	94.2%	4.0%
	HQC	250.00	103.3%	5.6%	85.6%	5.9%
l-alanine	LQC	3.91	91.3%	7.4%	102.4%	9.7%
	MQC	125.00	107.1%	9.5%	105.8%	12.4%
	HQC	250.00	97.7%	6.5%	92.9%	3.3%
*trans*-4-hydroxy-l-proline	LQC	1.95	97.2%	4.0%	99.9%	9.7%
	MQC	125.00	107.5%	6.6%	96.5%	5.7%
	HQC	250.00	102.3%	7.0%	97.1%	4.8%
l-threonine	LQC	1.95	86.3%	6.4%	93.1%	11.8%
	MQC	125.00	102.6%	4.7%	98.8%	9.1%
	HQC	250.00	109.1%	4.5%	95.2%	4.0%
l-glycine	LQC	173.44	97.7%	7.7%	93.9%	8.6%
	MQC	5550.00	102.1%	9.2%	96.7%	5.9%
	HQC	11,100.00	107.4%	3.5%	91.3%	5.6%
l-glutamine	LQC	1.73	85.0%	5.1%	99.9%	4.3%
	MQC	111.00	105.2%	3.4%	106.3%	6.9%
	HQC	222.00	100.7%	5.0%	101.3%	6.4%
l-serine	LQC	1.95	93.0%	9.0%	95.2%	8.6%
	MQC	125.00	96.2%	4.9%	94.1%	10.0%
	HQC	250.00	98.2%	4.8%	92.7%	5.2%
l-asparagine	LQC	1.73	87.0%	7.1%	103.2%	9.3%
	MQC	111.00	103.3%	2.4%	103.0%	13.6%
	HQC	222.00	106.4%	6.1%	97.5%	5.2%
l-glutamic acid	LQC	1.95	90.4%	8.4%	96.8%	7.3%
	MQC	125.00	100.6%	6.4%	101.0%	4.6%
	HQC	250.00	106.7%	3.5%	92.1%	3.9%
l-citrulline	LQC	1.95	107.7%	8.2%	117.5%	10.4%
	MQC	125.00	102.8%	8.0%	97.5%	7.7%
	HQC	250.00	97.7%	2.1%	92.3%	4.1%
1-methyl-l-histidine	LQC	1.95	90.4%	8.0%	108.4%	5.6%
	MQC	125.00	112.1%	7.1%	95.0%	8.7%
	HQC	250.00	98.1%	4.7%	96.8%	1.6%
l-aspartic acid	LQC	3.91	94.9%	10.2%	89.5%	12.5%
	MQC	125.00	97.6%	9.0%	94.5%	9.1%
	HQC	250.00	108.8%	6.6%	93.3%	8.7%
l-histidine	LQC	3.91	97.6%	10.3%	100.9%	5.9%
	MQC	125.00	104.0%	7.1%	90.5%	8.6%
	HQC	250.00	110.1%	4.2%	98.1%	4.8%
3-methyl-l-histidine	LQC	1.95	93.1%	9.8%	111.4%	9.4%
	MQC	125.00	107.7%	5.0%	93.5%	4.1%
	HQC	250.00	104.2%	2.8%	87.8%	2.6%
l-arginine	LQC	1.95	98.6%	11.5%	90.4%	7.2%
	MQC	125.00	103.5%	5.5%	97.1%	5.9%
	HQC	250.00	105.3%	1.9%	94.4%	2.5%
l-lysine	LQC	1.95	94.5%	8.1%	91.3%	11.6%
	MQC	125.00	105.6%	2.4%	101.2%	4.4%
	HQC	250.00	98.0%	4.9%	87.3%	3.6%
homocystine	LQC	3.91	89.7%	6.1%	104.8%	9.8%
	MQC	125.00	106.9%	4.1%	117.1%	8.6%
	HQC	250.00	103.4%	2.1%	107.4%	4.5%
l-ornithine	LQC	1.95	90.6%	8.2%	87.4%	13.6%
	MQC	125.00	108.8%	7.0%	99.8%	5.6%
	HQC	250.00	95.8%	3.0%	86.4%	4.2%
anserine	LQC	1.95	103.4%	8.3%	92.7%	6.4%
	MQC	125.00	111.1%	5.9%	87.5%	5.9%
	HQC	250.00	105.8%	3.7%	102.6%	4.8%
carnosine	LQC	1.95	97.3%	5.9%	96.7%	9.1%
	MQC	125.00	112.7%	7.0%	94.7%	9.1%
	HQC	250.00	104.8%	3.1%	98.6%	3.1%
δ-hydroxylysine	LQC	7.81	96.2%	10.1%	97.6%	8.4%
	MQC	125.00	97.9%	5.8%	95.5%	9.6%
	HQC	250.00	101.1%	3.0%	89.9%	6.0%
cystathionine	LQC	3.91	87.3%	9.6%	97.1%	4.9%
	MQC	125.00	109.1%	6.1%	92.6%	7.8%
	HQC	250.00	101.1%	4.7%	90.6%	2.6%
l-cystine	LQC	1.95	107.3%	9.3%	89.1%	9.5%
	MQC	125.00	100.6%	6.6%	86.8%	9.5%
	HQC	250.00	104.4%	4.7%	84.8%	4.9%

**Table 4:** Recovery and matrix effects of the analyzed AAs and their related compounds.

Amino Acids and Related Compounds		Concentration (μM)	Recovery (%)	RSD (%)	Matrix Effects (%)	RSD (%)
*N*-acetyl-l-cysteine	LQC	1.73	101.9%	4.5%	168.3%	15.6%
	MQC	111.00	89.4%	7.5%	140.9%	8.9%
	HQC	222.00	106.7%	4.9%	175.6%	4.5%
creatinine	LQC	1.95	90.6%	7.0%	124.8%	4.7%
	MQC	125.00	86.3%	6.0%	134.0%	5.9%
	HQC	250.00	104.4%	5.3%	155.5%	3.6%
3-cyclohexyl-d-alanine hydrate	LQC	0.87	101.6%	8.8%	93.1%	4.5%
	MQC	55.50	95.8%	7.0%	106.1%	7.0%
	HQC	111.00	90.4%	6.2%	99.3%	4.6%
3-nitro-l-tyrosine	LQC	3.47	110.0%	7.4%	167.7%	9.2%
	MQC	111.00	116.1%	13.8%	130.5%	7.7%
	HQC	222.00	91.4%	5.0%	151.2%	9.5%
l-phenylalanine	LQC	1.95	85.0%	3.2%	104.5%	14.9%
	MQC	125.00	91.6%	7.5%	95.8%	4.7%
	HQC	250.00	83.4%	5.0%	101.2%	3.9%
l-tryptophan	LQC	3.91	107.0%	6.3%	92.6%	5.2%
	MQC	125.00	101.3%	5.4%	104.3%	4.5%
	HQC	250.00	88.6%	2.6%	90.1%	1.6%
l-5-oxoproline	LQC	5.78	87.9%	6.9%	180.4%	9.0%
	MQC	1110.00	103.9%	7.3%	159.0%	11.8%
	HQC	2220.00	98.3%	5.2%	169.0%	5.2%
kynurenine	LQC	1.73	85.3%	7.2%	162.2%	7.6%
	MQC	55.50	96.3%	2.8%	174.8%	5.8%
	HQC	111.00	91.6%	5.8%	141.6%	5.6%
l-leucine	LQC	1.95	111.2%	9.4%	115.6%	5.5%
	MQC	125.00	116.9%	5.3%	122.6%	6.1%
	HQC	250.00	99.5%	8.0%	101.5%	4.5%
l-isoleucine	LQC	3.91	86.4%	6.7%	198.7%	6.9%
	MQC	125.00	93.6%	4.5%	184.2%	5.8%
	HQC	250.00	114.4%	2.6%	160.5%	7.4%
l-(+)-alpha-phenylglycine	LQC	1.73	118.1%	7.4%	168.7%	12.9%
	MQC	111.00	98.7%	6.1%	186.1%	4.9%
	HQC	222.00	104.4%	3.9%	196.5%	5.0%
ethanolamine	LQC	1.95	96.0%	7.2%	178.5%	5.5%
	MQC	125.00	97.2%	4.8%	184.4%	5.8%
	HQC	250.00	87.2%	3.0%	160.0%	5.7%
(*R*)-(−)-2-(2,5-dihydrophenyl)glycine	LQC	1.73	110.1%	9.9%	200.1%	12.3%
	MQC	111.00	105.4%	7.7%	189.3%	3.8%
	HQC	222.00	93.2%	4.5%	184.7%	5.4%
l-methionine	LQC	3.91	104.3%	4.4%	112.9%	2.9%
	MQC	125.00	85.4%	11.0%	108.8%	12.0%
	HQC	250.00	100.8%	7.6%	106.4%	10.7%
norvaline	LQC	1.73	98.0%	9.5%	172.5%	14.0%
	MQC	111.00	96.5%	8.5%	190.9%	9.4%
	HQC	222.00	103.3%	3.7%	166.9%	9.1%
γ-aminobutyric acid	LQC	3.91	111.7%	4.6%	140.1%	3.8%
	MQC	125.00	96.7%	6.2%	127.8%	6.5%
	HQC	250.00	95.6%	2.2%	131.4%	4.5%
l-valine	LQC	1.95	84.2%	4.9%	101.2%	11.0%
	MQC	125.00	81.7%	8.6%	105.3%	7.9%
	HQC	250.00	85.9%	3.0%	97.1%	13.6%
l-proline	LQC	1.95	93.8%	13.8%	101.4%	7.9%
	MQC	125.00	83.8%	6.4%	106.9%	12.9%
	HQC	250.00	87.1%	8.5%	111.7%	8.8%
dl-β-aminoisobutyric acid	LQC	7.81	90.1%	13.7%	171.0%	15.0%
	MQC	125.00	88.0%	10.3%	201.6%	13.2%
	HQC	250.00	98.2%	9.1%	195.0%	6.0%
l-tyrosine	LQC	3.91	91.1%	6.1%	112.1%	9.5%
	MQC	125.00	96.2%	3.0%	111.7%	5.9%
	HQC	250.00	88.3%	4.0%	107.7%	2.6%
l-taurine	LQC	3.47	98.4%	7.0%	206.2%	3.2%
	MQC	111.00	94.9%	2.2%	185.1%	9.3%
	HQC	222.00	101.4%	6.6%	194.7%	8.2%
l-cysteine	LQC	69.38	100.9%	6.8%	134.4%	6.1%
	MQC	1110.00	102.5%	5.0%	137.8%	4.1%
	HQC	2220.00	86.4%	4.4%	157.6%	7.7%
methionine sulfone	LQC	6.94	98.2%	6.2%	158.4%	14.5%
	MQC	111.00	102.9%	7.1%	142.6%	5.7%
	HQC	222.00	85.1%	6.8%	173.8%	2.9%
l-α-amino-n-butyric acid	LQC	1.95	96.7%	8.9%	146.2%	6.5%
	MQC	125.00	97.6%	2.5%	160.9%	5.7%
	HQC	250.00	92.2%	5.9%	132.2%	2.1%
sarcosine	LQC	1.95	102.4%	8.8%	171.3%	5.4%
	MQC	125.00	94.1%	5.8%	152.3%	4.7%
	HQC	250.00	100.8%	7.6%	182.4%	6.4%
β-alanine	LQC	3.91	117.3%	8.4%	147.6%	7.4%
	MQC	125.00	86.7%	4.7%	184.7%	9.6%
	HQC	250.00	88.6%	4.4%	174.1%	6.5%
l-alanine	LQC	3.91	94.6%	8.0%	118.7%	11.4%
	MQC	125.00	103.7%	6.6%	110.0%	8.6%
	HQC	250.00	96.5%	1.9%	102.1%	5.0%
l-threonine	LQC	1.95	100.9%	11.4%	102.5%	10.7%
	MQC	125.00	104.3%	7.3%	114.7%	6.9%
	HQC	250.00	99.2%	5.4%	107.3%	5.3%
*trans*-4-hydroxy-l-proline	LQC	1.95	106.1%	14.1%	168.9%	5.6%
	MQC	125.00	101.9%	5.2%	191.5%	6.2%
	HQC	250.00	107.1%	8.5%	181.6%	5.4%
l-glycine	LQC	173.44	104.7%	15.8%	112.7%	14.7%
	MQC	5550.00	97.9%	7.6%	105.1%	9.3%
	HQC	11,100.00	94.6%	4.6%	114.1%	6.0%
l-glutamine	LQC	1.73	81.6%	7.2%	94.3%	4.7%
	MQC	111.00	93.1%	6.6%	104.0%	7.1%
	HQC	222.00	84.9%	4.9%	112.2%	7.8%
l-serine	LQC	1.95	83.3%	4.0%	98.6%	8.5%
	MQC	125.00	103.6%	4.1%	112.9%	11.9%
	HQC	250.00	100.5%	3.3%	104.3%	2.5%
l-asparagine	LQC	1.73	90.6%	5.8%	101.4%	5.7%
	MQC	111.00	85.8%	3.5%	112.3%	3.5%
	HQC	222.00	101.7%	7.1%	105.4%	4.7%
l-glutamic acid	LQC	1.95	84.6%	4.3%	112.8%	10.5%
	MQC	125.00	90.9%	6.0%	106.0%	5.7%
	HQC	250.00	88.0%	6.4%	99.0%	6.6%
l-citrulline	LQC	1.95	91.8%	6.1%	176.2%	2.9%
	MQC	125.00	85.8%	5.3%	162.5%	5.3%
	HQC	250.00	102.7%	4.0%	158.6%	6.3%
1-methyl-l-histidine	LQC	1.95	114.5%	8.0%	166.4%	4.9%
	MQC	125.00	88.5%	6.9%	177.4%	13.5%
	HQC	250.00	108.3%	4.9%	143.6%	4.4%
l-aspartic acid	LQC	3.91	94.2%	6.9%	105.3%	5.7%
	MQC	125.00	81.3%	10.0%	95.1%	5.9%
	HQC	250.00	99.2%	5.8%	87.2%	6.1%
l-histidine	LQC	3.91	103.2%	8.9%	127.1%	5.8%
	MQC	125.00	99.7%	6.7%	124.1%	3.7%
	HQC	250.00	92.2%	3.1%	137.1%	1.3%
3-methyl-l-histidine	LQC	1.95	109.1%	12.9%	164.4%	13.5%
	MQC	125.00	92.2%	5.7%	188.4%	5.9%
	HQC	250.00	104.2%	2.6%	141.6%	3.9%
l-arginine	LQC	1.95	95.7%	7.7%	97.4%	12.8%
	MQC	125.00	101.1%	2.8%	101.7%	7.0%
	HQC	250.00	87.5%	3.1%	105.8%	1.9%
l-lysine	LQC	1.95	89.4%	2.5%	105.1%	6.9%
	MQC	125.00	90.9%	7.7%	114.4%	14.4%
	HQC	250.00	88.2%	6.3%	109.2%	7.3%
homocystine	LQC	3.91	89.1%	12.3%	153.0%	8.6%
	MQC	125.00	104.9%	6.3%	169.5%	7.2%
	HQC	250.00	106.5%	5.1%	182.3%	7.5%
l-ornithine	LQC	1.95	89.1%	6.2%	155.2%	7.2%
	MQC	125.00	84.4%	5.6%	167.2%	6.5%
	HQC	250.00	90.9%	3.9%	189.4%	4.5%
anserine	LQC	1.95	96.1%	6.0%	166.8%	5.5%
	MQC	125.00	92.3%	3.7%	172.1%	6.6%
	HQC	250.00	104.9%	5.1%	141.4%	3.3%
carnosine	LQC	1.95	103.7%	7.9%	130.8%	4.9%
	MQC	125.00	101.8%	7.2%	151.9%	7.9%
	HQC	250.00	92.5%	4.9%	128.5%	9.6%
δ-hydroxylysine	LQC	7.81	103.1%	7.4%	94.7%	10.6%
	MQC	125.00	107.7%	8.1%	114.8%	6.9%
	HQC	250.00	85.6%	4.1%	110.8%	6.7%
cystathionine	LQC	3.91	100.1%	8.7%	127.1%	7.7%
	MQC	125.00	118.8%	6.1%	140.1%	5.7%
	HQC	250.00	94.2%	4.0%	161.3%	3.2%
l-cystine	LQC	1.95	110.5%	6.5%	100.8%	6.7%
	MQC	125.00	106.6%	2.5%	104.3%	2.9%
	HQC	250.00	82.4%	4.3%	109.5%	3.9%

**Table 5:** Stability and dilution integrity of the analyzed AAs and their related compounds.

Amino Acids and Related Compounds		Concentration (μM)	Stability	RSD (%)	Freeze–Thaw	RSD (%)	Dilution Factor (20)	
			4 °C		3 Cycles		Accuracy (%)	Precision (%)
*N*-acetyl-l-cysteine	LQC	1.73	99.5%	9.5%	94.6%	8.0%	96.5%	3.1%
	MQC	111.00	93.4%	12.6%	90.6%	6.5%		
	HQC	222.00	98.0%	1.5%	94.9%	2.1%		
creatinine	LQC	1.95	97.1%	9.2%	95.9%	6.9%	104.3%	8.8%
	MQC	125.00	91.6%	5.3%	100.6%	6.6%		
	HQC	250.00	105.2%	2.6%	95.2%	2.1%		
3-cyclohexyl-d-alanine hydrate	LQC	0.87	103.6%	5.1%	99.1%	9.2%	97.2%	4.6%
	MQC	55.50	85.2%	3.9%	104.8%	7.4%		
	HQC	111.00	86.9%	1.9%	90.8%	4.8%		
3-nitro-l-tyrosine	LQC	3.47	92.9%	9.2%	101.7%	14.3%	106.2%	5.3%
	MQC	111.00	102.3%	5.6%	95.2%	7.4%		
	HQC	222.00	96.4%	4.7%	92.2%	3.6%		
l-phenylalanine	LQC	1.95	99.6%	9.5%	92.7%	7.4%	98.4%	7.3%
	MQC	125.00	105.0%	4.1%	99.6%	3.0%		
	HQC	250.00	92.0%	6.0%	99.8%	2.5%		
l-tryptophan	LQC	3.91	102.0%	9.6%	90.0%	8.7%	97.1%	6.0%
	MQC	125.00	108.0%	4.1%	104.5%	6.4%		
	HQC	250.00	87.3%	6.1%	90.2%	5.5%		
l-5-oxoproline	LQC	5.78	91.1%	9.4%	104.2%	10.5%	92.9%	3.1%
	MQC	1110.00	87.8%	9.6%	90.7%	7.5%		
	HQC	2220.00	102.9%	5.8%	95.3%	5.0%		
kynurenine	LQC	1.73	101.3%	7.9%	99.4%	10.5%	98.7%	8.6%
	MQC	55.50	118.7%	7.6%	111.6%	6.0%		
	HQC	111.00	104.4%	5.6%	87.3%	4.1%		
l-leucine	LQC	1.95	90.9%	8.3%	99.7%	7.0%	94.3%	8.2%
	MQC	125.00	87.7%	9.4%	98.5%	5.3%		
	HQC	250.00	102.6%	4.7%	97.7%	2.9%		
l-isoleucine	LQC	3.91	102.9%	8.1%	104.4%	7.6%	96.4%	3.3%
	MQC	125.00	107.5%	3.6%	90.4%	7.0%		
	HQC	250.00	99.2%	4.9%	97.2%	5.1%		
l-(+)-alpha-phenylglycine	LQC	1.73	90.1%	9.2%	98.9%	10.7%	93.7%	2.6%
	MQC	111.00	95.6%	8.7%	94.3%	5.0%		
	HQC	222.00	89.1%	7.5%	84.2%	3.6%		
ethanolamine	LQC	1.95	107.0%	4.0%	104.6%	4.7%	92.9%	4.0%
	MQC	125.00	109.2%	7.4%	96.8%	6.4%		
	HQC	250.00	102.0%	5.8%	107.9%	5.7%		
(*R*)-(−)-2-(2,5-dihydrophenyl)glycine	LQC	1.73	94.2%	9.3%	95.9%	8.6%	96.9%	3.5%
	MQC	111.00	92.3%	4.5%	86.1%	11.1%		
	HQC	222.00	101.2%	3.5%	99.5%	5.2%		
l-methionine	LQC	3.91	94.5%	8.0%	98.2%	5.9%	103.0%	6.4%
	MQC	125.00	91.3%	4.9%	95.7%	4.9%		
	HQC	250.00	90.0%	4.1%	86.4%	1.3%		
norvaline	LQC	1.73	101.6%	9.9%	90.1%	8.0%	102.0%	5.5%
	MQC	111.00	97.0%	7.2%	95.6%	3.7%		
	HQC	222.00	101.2%	4.7%	87.9%	2.3%		
γ-aminobutyric acid	LQC	3.91	90.4%	5.3%	88.8%	13.6%	96.2%	8.5%
	MQC	125.00	97.7%	6.0%	96.7%	2.0%		
	HQC	250.00	108.2%	7.5%	100.1%	4.7%		
l-valine	LQC	1.95	100.4%	6.1%	92.8%	5.8%	95.7%	4.2%
	MQC	125.00	97.4%	5.1%	90.5%	5.7%		
	HQC	250.00	93.9%	6.4%	87.1%	2.3%		
l-proline	LQC	1.95	91.5%	8.0%	99.2%	12.1%	97.3%	7.9%
	MQC	125.00	87.0%	6.6%	96.9%	6.7%		
	HQC	250.00	94.9%	5.8%	97.6%	3.2%		
dl-β-aminoisobutyric acid	LQC	7.81	100.2%	9.4%	99.2%	9.2%	96.3%	6.1%
	MQC	125.00	92.1%	6.8%	95.7%	7.7%		
	HQC	250.00	99.5%	3.7%	102.1%	3.0%		
l-tyrosine	LQC	3.91	99.2%	13.5%	106.4%	4.9%	94.7%	3.2%
	MQC	125.00	107.8%	5.5%	115.1%	10.1%		
	HQC	250.00	101.8%	5.0%	108.2%	4.1%		
l-taurine	LQC	3.47	88.5%	4.3%	93.3%	8.8%	97.1%	2.0%
	MQC	111.00	98.1%	8.7%	97.8%	8.1%		
	HQC	222.00	108.9%	4.7%	102.4%	11.9%		
l-cysteine	LQC	69.38	100.1%	8.6%	98.6%	9.8%	89.7%	7.5%
	MQC	1110.00	95.3%	5.9%	108.2%	6.2%		
	HQC	2220.00	94.0%	3.6%	84.0%	3.7%		
methionine sulfone	LQC	6.94	94.3%	13.9%	94.7%	9.6%	95.4%	5.6%
	MQC	111.00	91.4%	5.6%	98.3%	9.3%		
	HQC	222.00	98.2%	4.4%	100.8%	6.0%		
l-α-amino-n-butyric acid	LQC	1.95	95.3%	11.1%	93.1%	15.4%	100.9%	4.7%
	MQC	125.00	99.9%	5.8%	98.6%	3.3%		
	HQC	250.00	91.2%	4.6%	101.0%	3.8%		
sarcosine	LQC	1.95	104.8%	11.3%	94.0%	11.0%	105.6%	2.8%
	MQC	125.00	90.0%	7.5%	102.2%	4.6%		
	HQC	250.00	93.4%	5.4%	99.2%	2.4%		
β-alanine	LQC	3.91	103.0%	13.1%	86.8%	3.9%	86.9%	4.7%
	MQC	125.00	100.7%	4.9%	96.0%	6.3%		
	HQC	250.00	99.0%	3.5%	102.4%	4.7%		
l-alanine	LQC	3.91	116.1%	13.1%	92.4%	10.1%	91.4%	4.0%
	MQC	125.00	102.4%	9.3%	88.2%	6.4%		
	HQC	250.00	97.2%	5.0%	113.5%	3.8%		
l-threonine	LQC	1.95	100.6%	5.9%	104.4%	6.0%	95.6%	5.8%
	MQC	125.00	103.3%	3.4%	102.6%	5.8%		
	HQC	250.00	102.0%	3.8%	98.3%	3.4%		
*trans*-4-hydroxy-l-proline	LQC	1.95	93.0%	3.4%	92.9%	7.2%	96.6%	5.2%
	MQC	125.00	96.2%	9.0%	98.1%	6.4%		
	HQC	250.00	90.2%	5.0%	86.4%	3.7%		
l-glycine	LQC	173.44	96.4%	7.9%	83.5%	8.5%	98.6%	5.2%
	MQC	5550.00	86.7%	4.2%	89.2%	6.8%		
	HQC	11,100.00	92.3%	4.3%	95.2%	3.2%		
l-glutamine	LQC	1.73	95.5%	12.3%	114.9%	4.8%	101.7%	4.6%
	MQC	111.00	117.9%	7.5%	106.9%	6.2%		
	HQC	222.00	97.5%	6.6%	102.5%	4.8%		
l-serine	LQC	1.95	92.8%	6.0%	94.2%	8.8%	97.1%	6.3%
	MQC	125.00	105.2%	6.6%	97.1%	6.5%		
	HQC	250.00	88.8%	5.9%	93.4%	4.2%		
l-asparagine	LQC	1.73	89.3%	11.1%	104.5%	5.7%	102.0%	6.0%
	MQC	111.00	105.8%	10.8%	97.7%	4.7%		
	HQC	222.00	99.9%	5.0%	99.6%	1.4%		
l-glutamic acid	LQC	1.95	96.8%	6.4%	87.5%	5.1%	92.1%	3.9%
	MQC	125.00	91.3%	5.9%	90.4%	2.6%		
	HQC	250.00	94.7%	4.8%	91.6%	1.5%		
l-citrulline	LQC	1.95	93.1%	5.5%	95.4%	8.2%	98.4%	3.3%
	MQC	125.00	99.3%	4.1%	99.8%	6.0%		
	HQC	250.00	99.2%	4.2%	102.9%	2.4%		
1-methyl-l-histidine	LQC	1.95	116.5%	4.5%	91.4%	6.5%	96.0%	5.7%
	MQC	125.00	93.9%	6.5%	98.3%	3.5%		
	HQC	250.00	102.8%	5.7%	93.2%	2.6%		
l-aspartic acid	LQC	3.91	94.4%	5.0%	90.5%	10.7%	94.1%	8.3%
	MQC	125.00	92.0%	6.3%	101.5%	6.3%		
	HQC	250.00	105.7%	5.3%	93.2%	1.8%		
l-histidine	LQC	3.91	98.0%	6.4%	104.6%	8.6%	98.0%	6.3%
	MQC	125.00	93.4%	8.6%	97.4%	1.8%		
	HQC	250.00	99.2%	5.2%	100.8%	1.7%		
3-methyl-l-histidine	LQC	1.95	99.9%	8.2%	105.8%	5.6%	93.0%	5.7%
	MQC	125.00	96.6%	7.8%	91.8%	3.6%		
	HQC	250.00	99.2%	5.1%	103.4%	3.2%		
l-arginine	LQC	1.95	101.9%	12.4%	104.8%	5.3%	98.7%	2.4%
	MQC	125.00	108.0%	7.1%	109.0%	3.8%		
	HQC	250.00	110.0%	5.8%	99.4%	1.8%		
l-lysine	LQC	1.95	93.2%	12.3%	99.0%	7.3%	88.5%	2.4%
	MQC	125.00	90.9%	7.2%	95.2%	3.8%		
	HQC	250.00	102.2%	2.2%	89.8%	0.9%		
homocystine	LQC	3.91	95.2%	8.8%	101.7%	7.9%	99.7%	4.3%
	MQC	125.00	111.6%	2.6%	93.0%	5.5%		
	HQC	250.00	98.0%	5.3%	94.8%	4.0%		
l-ornithine	LQC	1.95	113.0%	12.2%	104.2%	9.4%	93.9%	7.5%
	MQC	125.00	98.7%	8.3%	93.0%	7.4%		
	HQC	250.00	84.3%	1.6%	96.8%	2.6%		
anserine	LQC	1.95	95.8%	10.0%	96.3%	7.9%	94.4%	6.4%
	MQC	125.00	92.2%	2.4%	101.3%	4.3%		
	HQC	250.00	99.7%	6.8%	98.5%	3.1%		
carnosine	LQC	1.95	93.2%	6.2%	98.0%	8.2%	98.4%	3.6%
	MQC	125.00	101.9%	5.4%	100.4%	4.4%		
	HQC	250.00	94.0%	4.5%	96.0%	3.9%		
δ-hydroxylysine	LQC	7.81	98.4%	8.1%	85.6%	5.2%	89.8%	4.7%
	MQC	125.00	95.8%	7.4%	101.8%	3.7%		
	HQC	250.00	90.9%	4.5%	84.1%	3.4%		
cystathionine	LQC	3.91	113.1%	8.2%	105.9%	7.9%	101.9%	14.0%
	MQC	125.00	86.5%	7.0%	97.0%	6.5%		
	HQC	250.00	98.0%	3.3%	103.9%	1.4%		
l-cystine	LQC	1.95	91.4%	6.2%	89.5%	4.6%	95.6%	4.5%
	MQC	125.00	87.8%	3.2%	95.3%	3.1%		
	HQC	250.00	91.4%	3.8%	97.9%	2.9%		

## Data Availability

The raw data supporting the conclusions of this article will be made available by the authors upon request.

## Supplementary Materials

The following supporting information can be downloaded at: https://www.mdpi.com/article/10.3390/molecules29245993/s1. Table S1. The linearity groups. Figure S1. The TIC (Total Ion Chromatogram) of the two chromatographic columns and the extracted ion chromatograms (XIC) for cystine and cysteine. Figure S2. (a) Separation of five pairs of isomers in the presence of 0.1% FA. (b) Separation of five pairs of isomers at the concentration 0.15% FA. (c) Separation of five pairs of isomers at the concentration 0.2% FA. Figure S3. (a) Signal of cysteine and glycine in the presence of only AMF (10 mM). (b) Signal of cysteine and glycine in the presence of only AMF (5 mM). (c) Signal of cysteine and glycine in the presence of only AMF (20 mM). Figure S4. Separation of AAs and related compounds under neutral and alkaline conditions. Figure S5. Separation of isomers 3-Methyl-l-histidine and 1-Methyl-l-histidine at about pH 3. Figure S6. The extraction response graph of AAs from plasma for different solvents, all containing 0.1% FA. Figure S7. (a) Represents the total MRM (Multiple Reaction Monitoring) channel after 100 mg of activated carbon was added to 1 mL of plasma and reacted for 8 h. (b) Represents the valine XIC (extracted ion chromatogram) under 100 mg of activated carbon, 8 h reaction. (c) Represents the proline XIC under 100 mg of activated carbon, 8 h reaction. (d) Represents the total MRM channel after 200 mg of activated carbon was added to 1 mL of plasma and reacted for 24 h. (e) Represents the valine XIC after 200 mg of activated carbon and a 24 h reaction. (f) Represents the proline XIC under 200 mg of activated carbon, 24 h reaction. Figure S8. The Total Ion Chromatogram of 48 AAs and their related compounds.
