# Supplementary material for: Analysis Profiling of 48 Endogenous Amino Acids and Related Compounds in Human Plasma Using Hydrophilic Interaction Liquid Chromatography–Tandem Mass Spectrometry
Source: Molecules. 2024 Dec 19;29(24):5993. doi: 10.3390/molecules29245993 (PMC11678698; doi:10.3390/molecules29245993)

Table S1. The linearity groups.

Group	AAs	Linearity (μM)	HQC (μM)	MQC (μM)	LQC (μM)
A	L-glycine	11100,5550,2775,1387.5, 693.75, 346.86,173.44.	11100	5550	173.44
B	kynurenine, 3-cyclohexyl-D-alanine	111,55.5,27.75,13.86, 6.94,3.49,1.73.	111	55.5	1.73
C	L-5-oxoproline, cysteine	2220, 1110,555, 138.75,69.38,34.69,17.34.	2220	1110	L-5-oxoproline, 17.34<br>cysteine, 69.38
D	asparagine, n-acetyl-l-cysteine, norvaline, methionine sulfone, 3-nitro-l-tyrosine, taurine, l-glutamine	222,111,55.5,27.75,13.88, 6.94, 3.47,1.73.	222	111	1.73 asparagine, n-acetyl-l-cysteine, norvaline, l-glutamine.<br>3.47 taurine, 3-nitro-L-tyrosine.<br>6.94 methionine sulfone.
E	L-anserine, L-alanine, L-arginine, L-aspartic acid, L-carnosine, L-creatinine, L-citrulline, L-cystine, cystathionine, ethanolamine, L-glutamic acid, L-histidine, L-isoleucine, L-leucine, L-lysine, L-methionine, L-phenylalanine, L-proline, L-serine, L-threonine, L-tyrosine, L-tryptophan, L-valine, β-alanine, L-α-amino-n-butyric acid, γ-amino-n-butyric acid, DL-β-aminoisobutyric acid, L-homocystine, δ-hydroxylysine, trans-4-hydroxy-L-proline, 1-methyl-L-histidine, 3-methyl-L-histidine, L-ornithine, sarcosine.	250, 125, 62.5, 31.25,15.63,7.81,3.91,1.95.	250	125	1.95 L-anserine, L-carnosine, L-creatinine, L-citrulline, L-cystine, L-leucine, L-lysine, ethanolamine, L-phenylalanine, L-proline, L-serine, L-threonine, L-valine, L-α-amino-n-butyric acid, 1-methyl-L-histidine, sarcosine, trans-4-hydroxy-L-proline, L-ornithine,3-methyl-L-histidine.<br>3.90 L-alanine, L-arginine, L-aspartic acid, cystathionine, L-glutamic acid, L-histidine, L-isoleucine, L-methionine. L-tyrosine, L-tryptophan, β-alanine, γ-amino-n-butyric acid, L-homocystine, δ-hydroxylysine.<br>7.81 DL-β-aminoisobutyric acid.

Figure S1. The TIC of the two chromatographic columns and the XIC for cystine and cysteine.

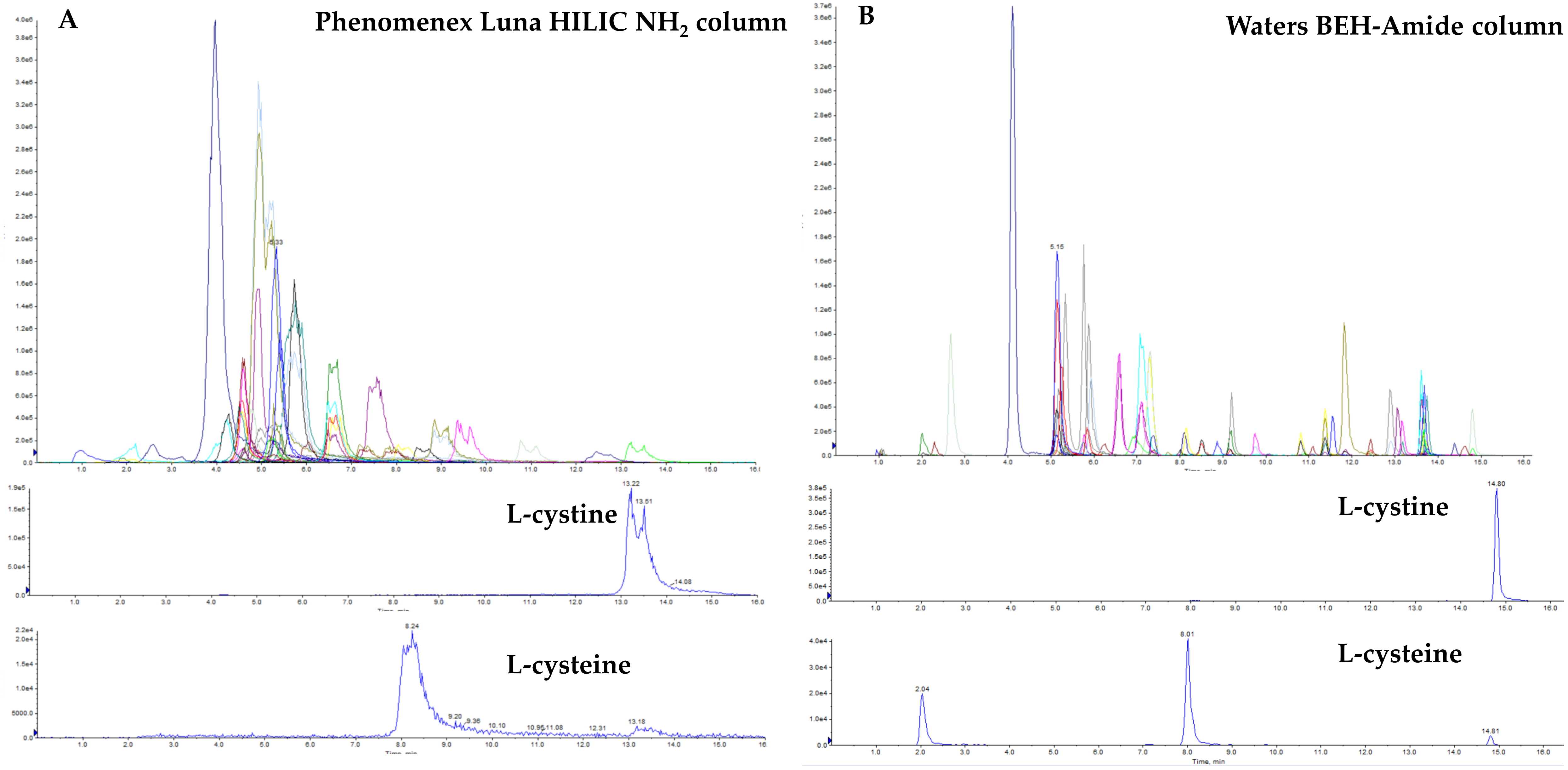

Figure S2. (a) Separation of Five Pairs of Isomers in the Presence of 0.1% FA.

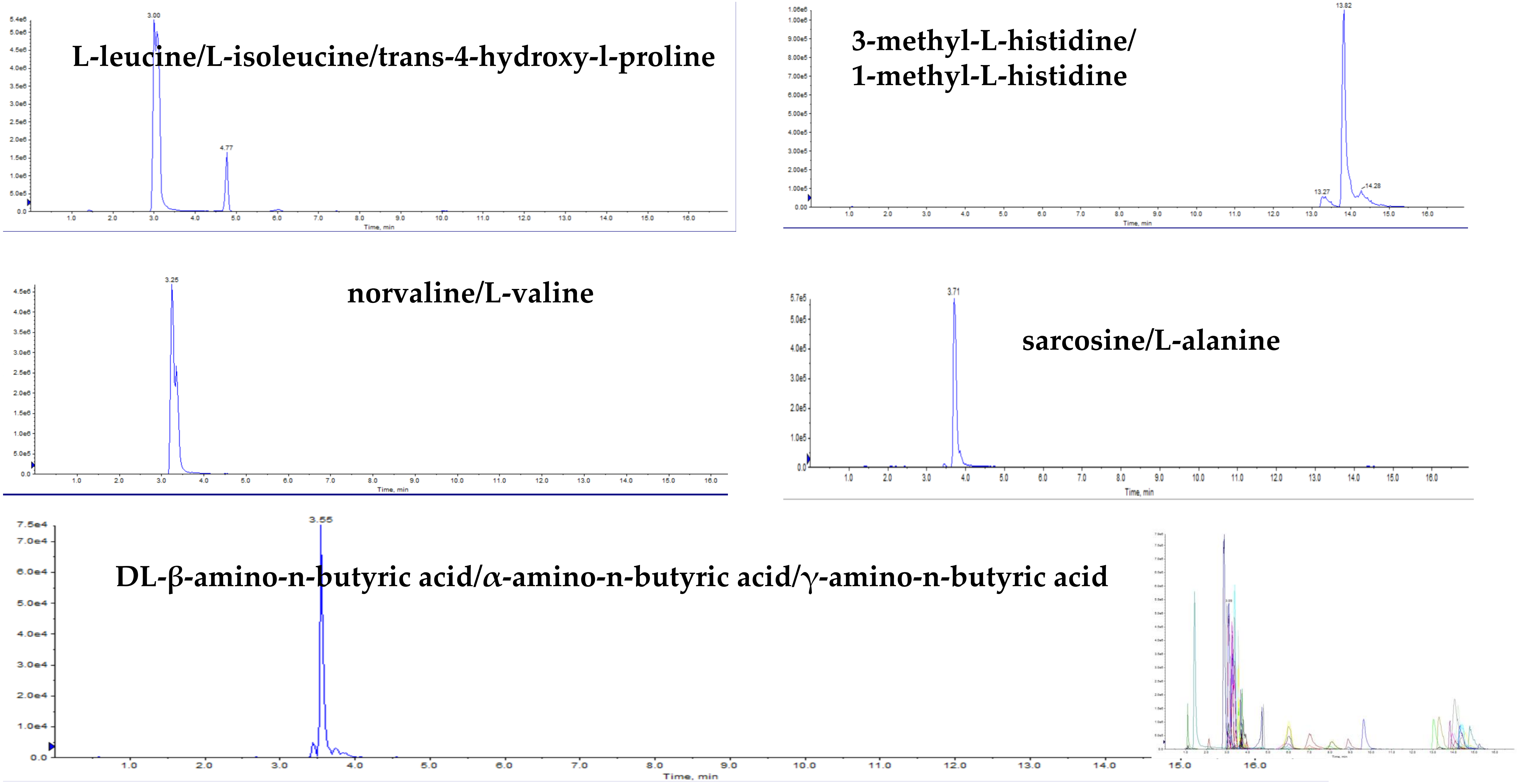

Figure S2. (b) Separation of Five Pairs of Isomers at the concentration of 0.15% FA.

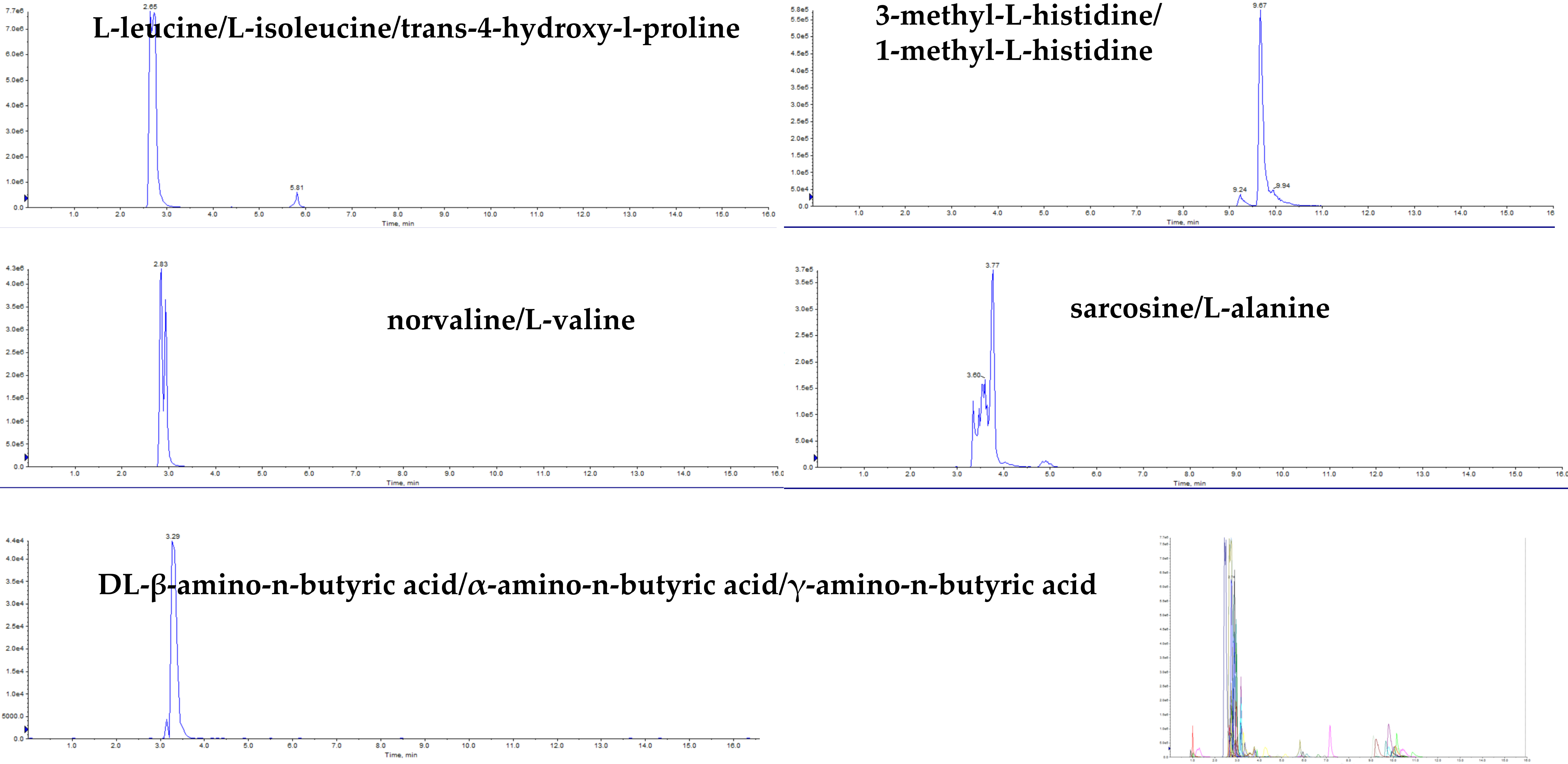

Figure S2. (c) Separation of Five Pairs of Isomers at the concentration of 0.2% FA.

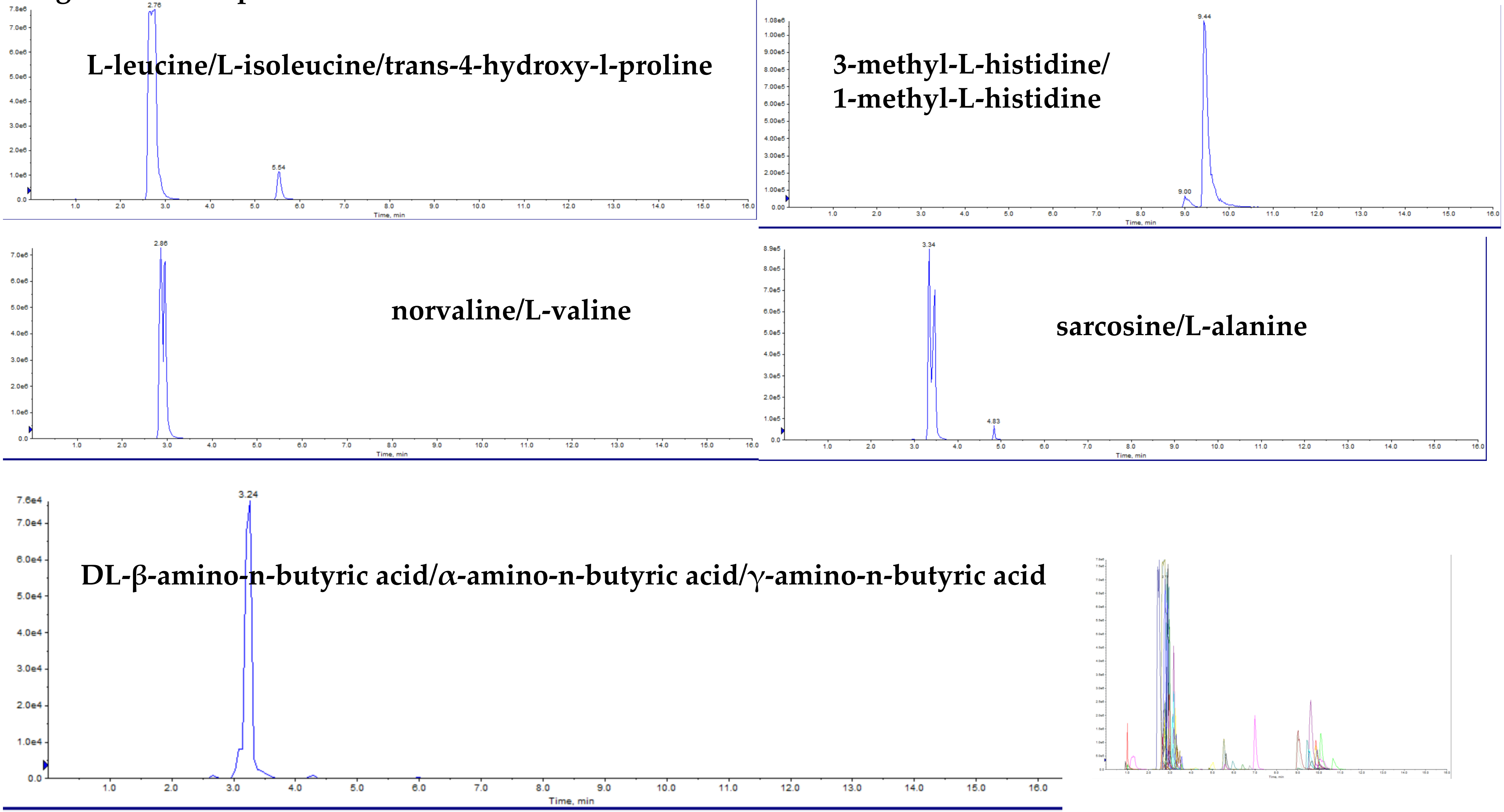

Figure S3. (a) Signal of L-cysteine and L-glycine in the presence of only AMF (10 mM).

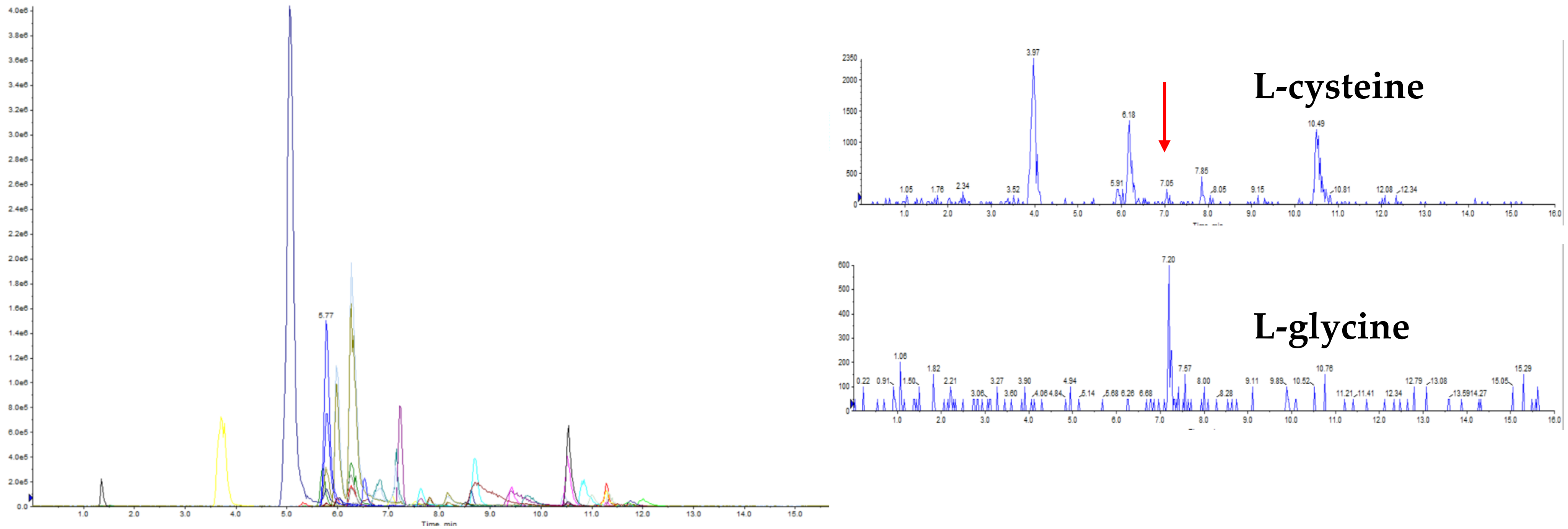

Figure S3. (b) Signal of L-cysteine and L-glycine in the presence of only AMF (5 mM).

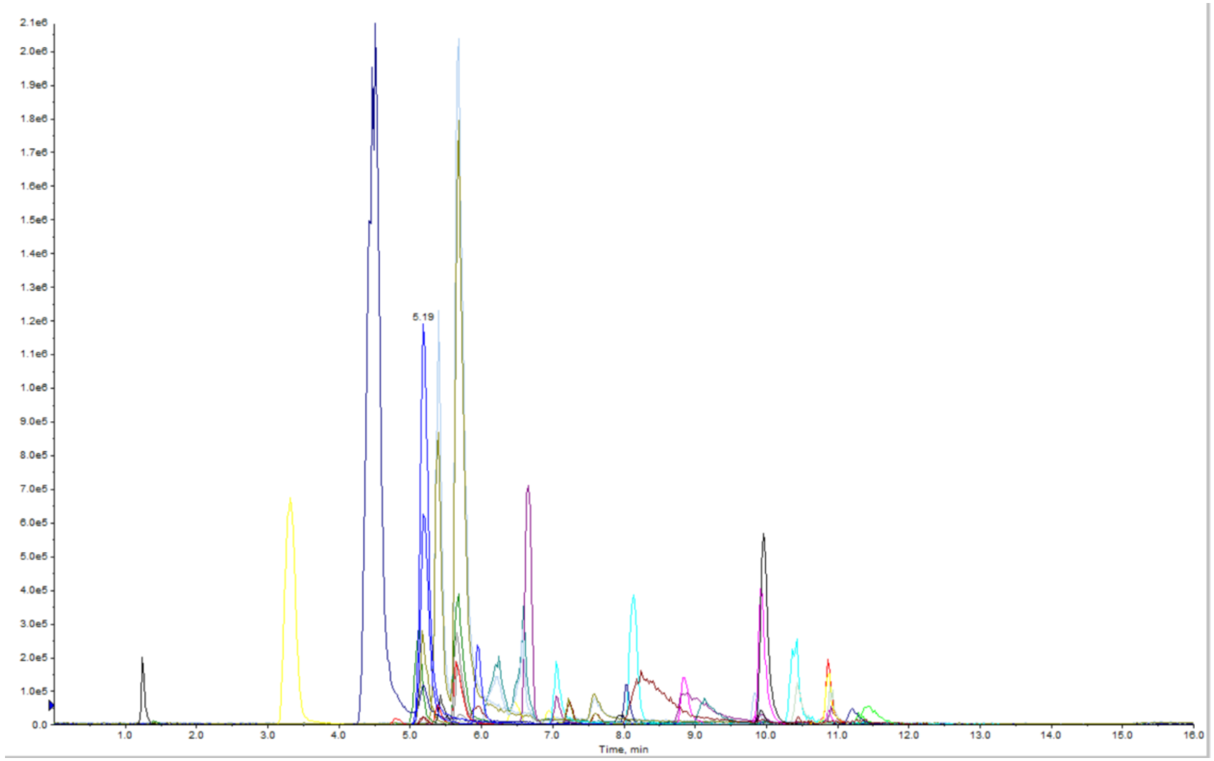

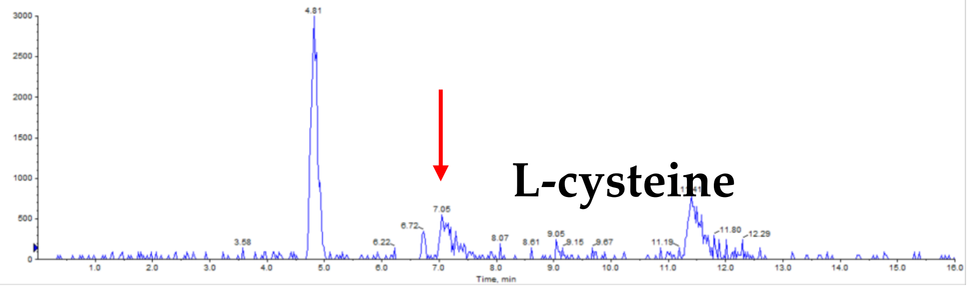

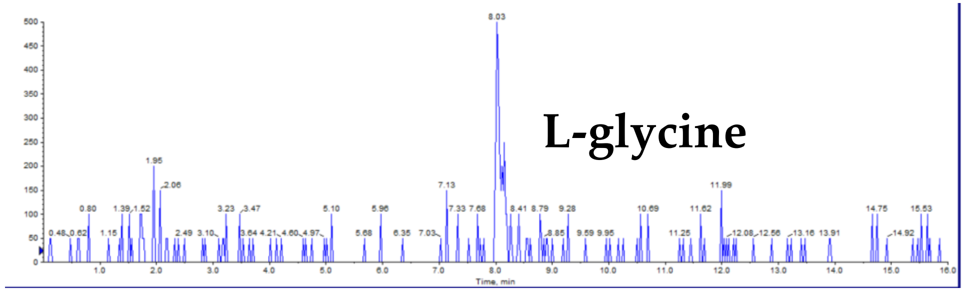

Figure S3. (c) Signal of L-cysteine and L-glycine in the presence of only AMF (20 mM).

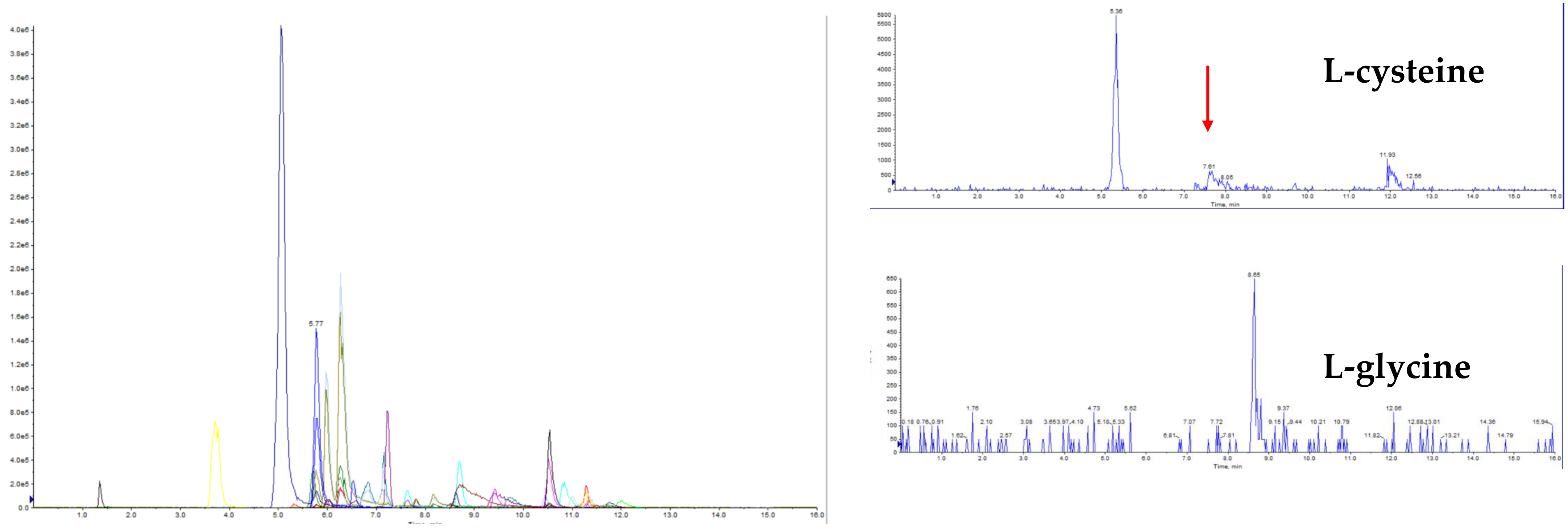

Figure S4. Separation of AAs and related compounds under neutral and alkaline conditions.

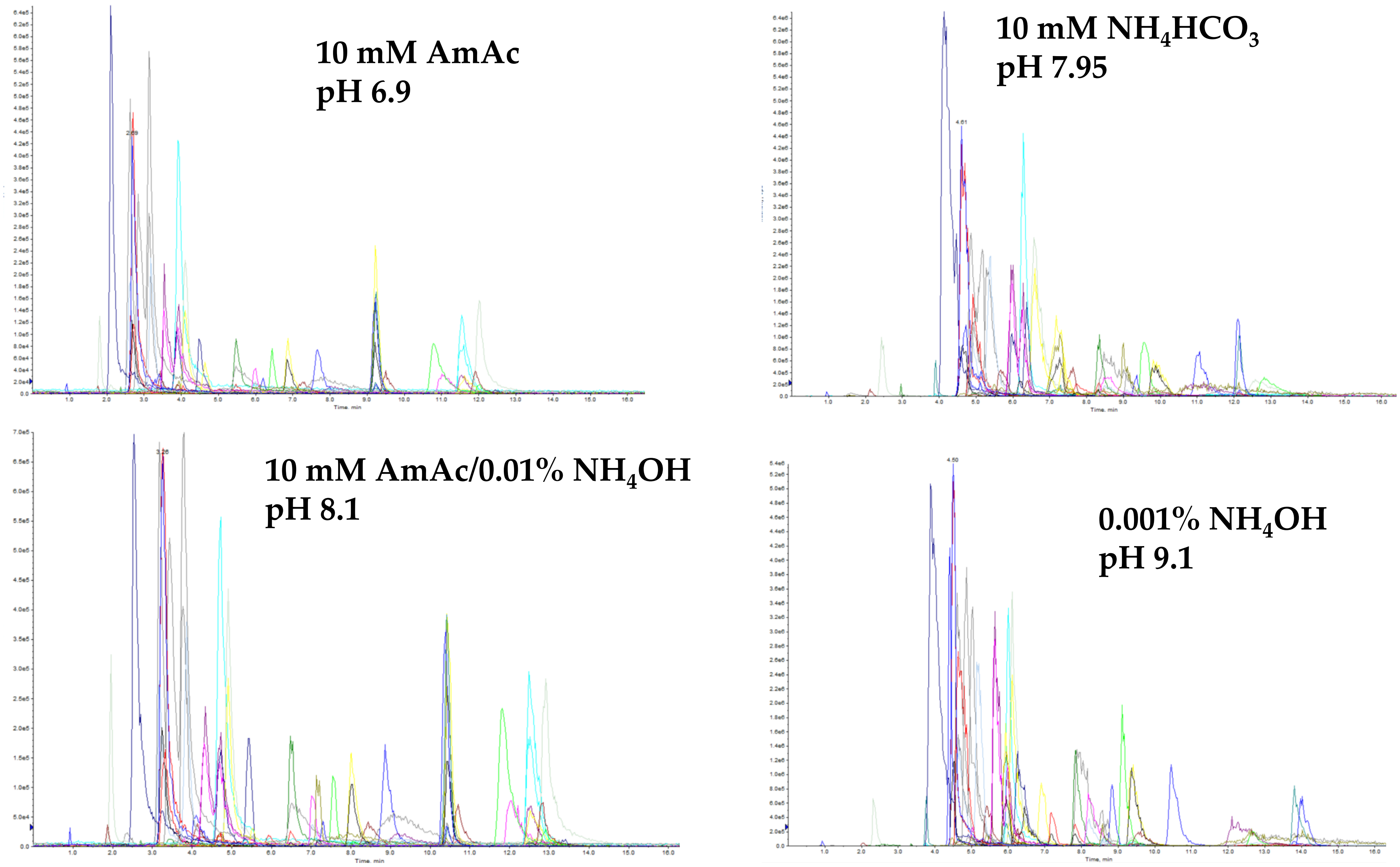

Figure S5. Separation of isomers 3-methyl-L-histidine and 1-methyl-L-histidine about pH 3.

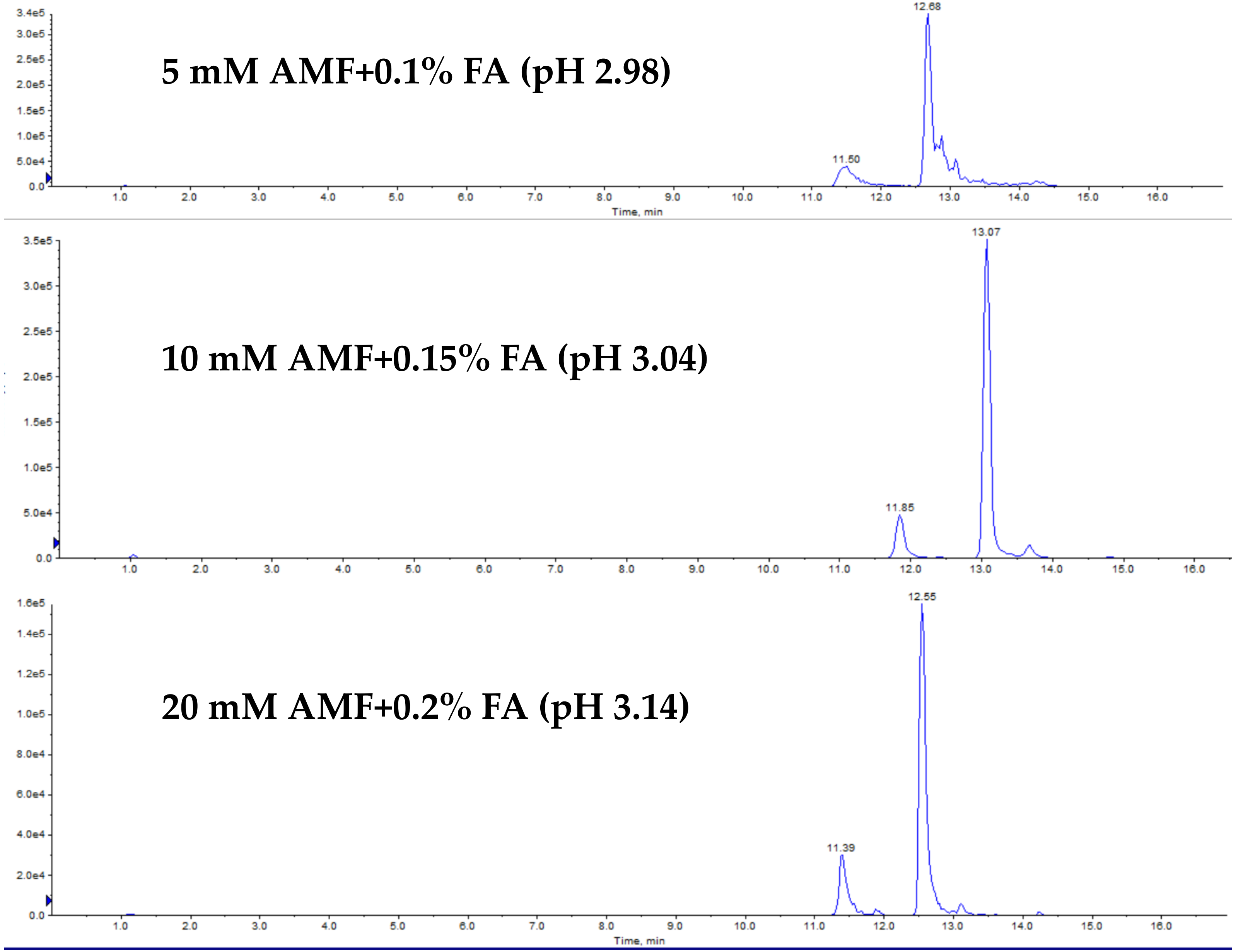

Figure S6. The extracted response graph of AAs from plasma for different solvents, all containing 0.1 % FA.

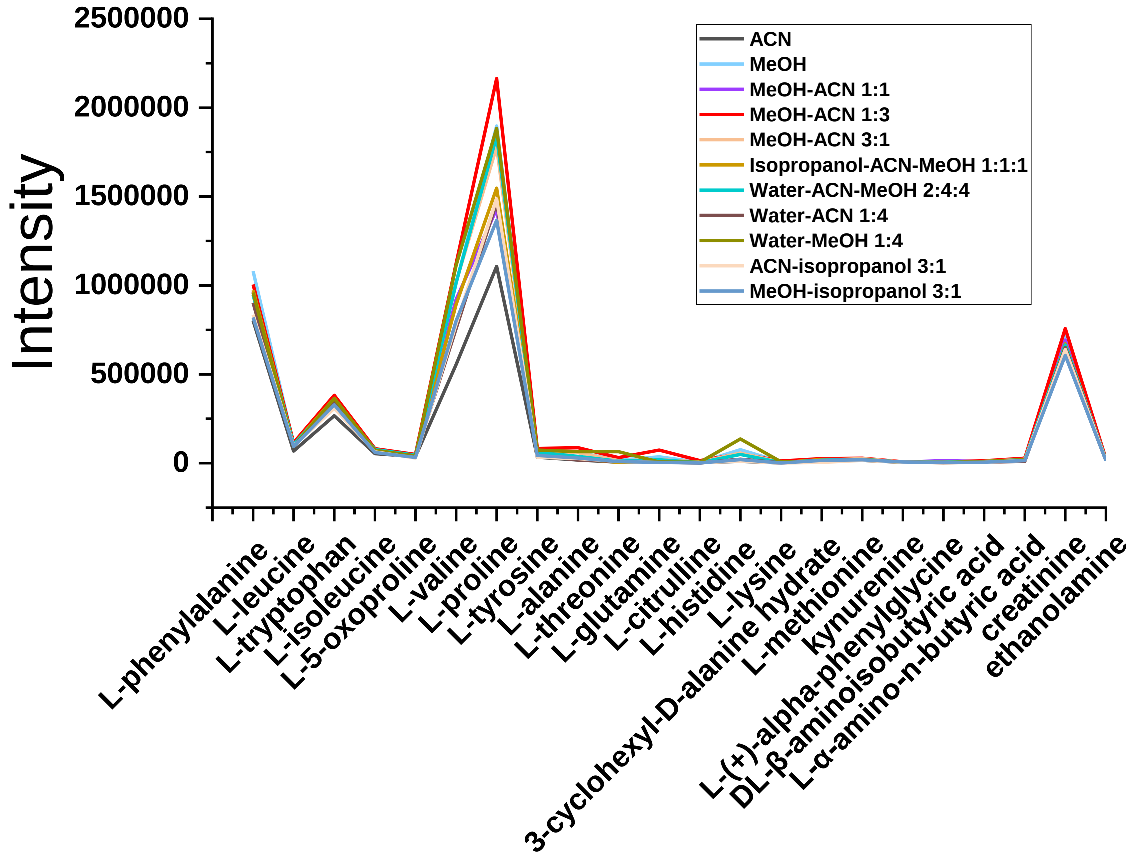

Figure S7. The TIC and XIC of the stripped plasma, a represents the total MRM (Multiple Reaction Monitoring) channel after 100 mg of activated carbon was added to 1 mL of plasma and reacted for 8 hours; **b** represents the valine XIC (Extracted Ion Chromatogram) under 100 mg activated carbon, 8 hours reaction; **c** represents the proline XIC under 100 mg activated carbon, 8 hours reaction; **d** represents the total MRM channel after 200 mg of activated carbon was added to 1 mL of plasma and reacted for 24 hours; **e** represents the valine XIC after 200 mg of activated carbon and a 24 hours reaction; **f** represents the proline XIC under 200 mg activated carbon, 24 hours reaction.

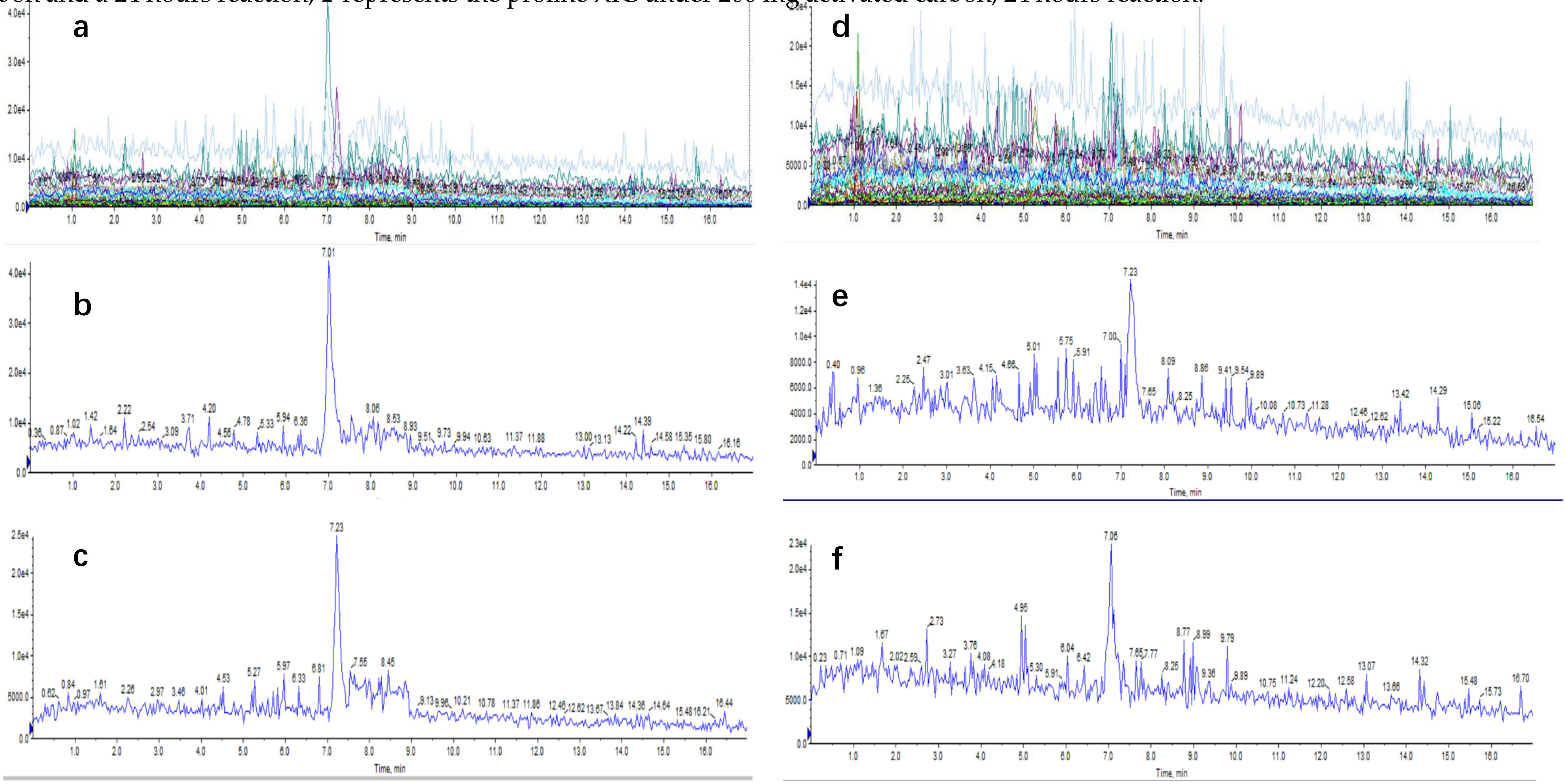

Figure S8. The total ion chromatogram of 48 AAs and their related compounds.

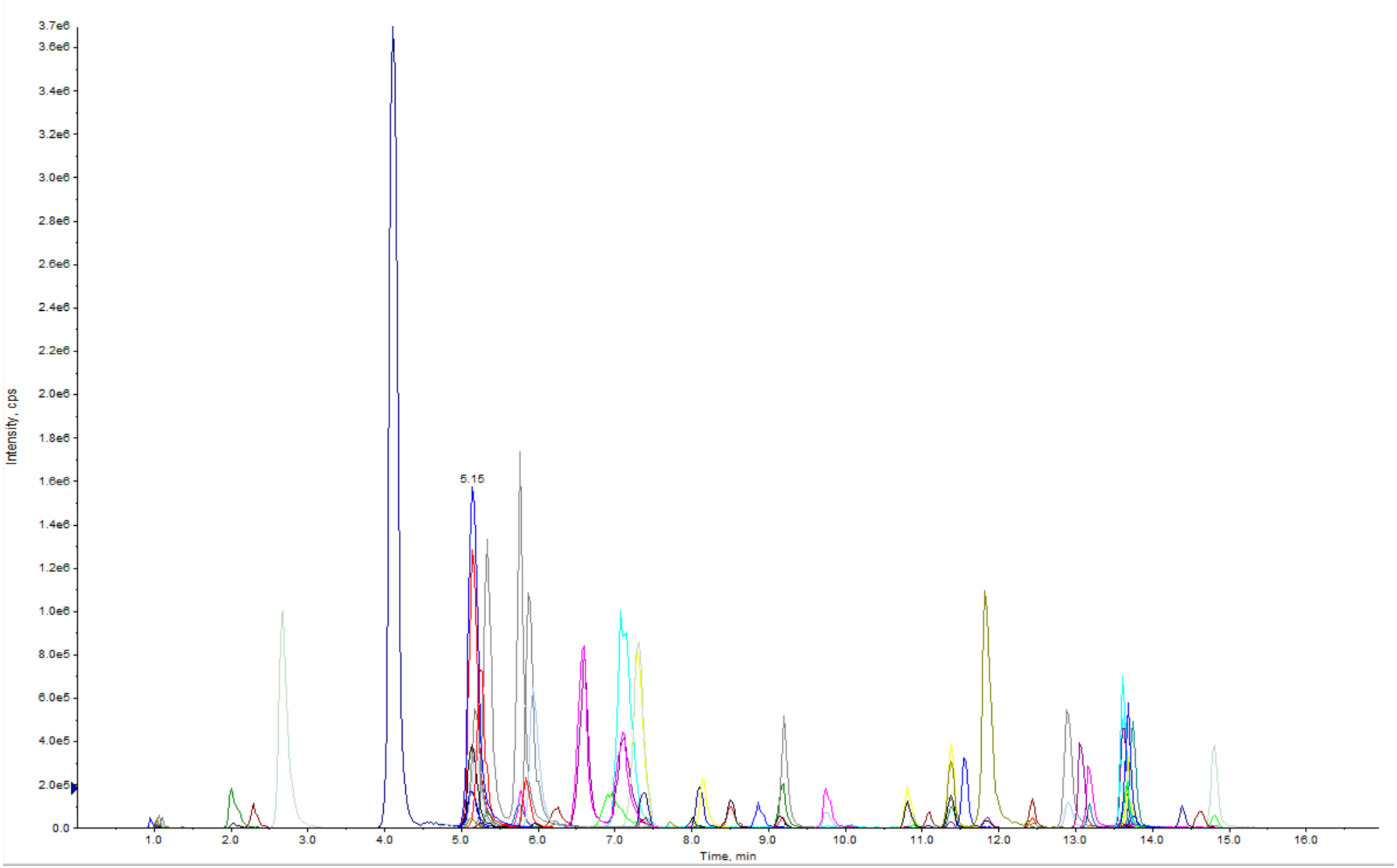

Supplement: Supplementary file 1 [file molecules-29-05993-s001.zip › molecules-3354382-supplementary.pdf]
